# Toward the automation of family semantic grids: a pilot exploration of text mining for evidence-informed systemic-relational psychotherapy

**DOI:** 10.3389/frcha.2026.1800848

**Published:** 2026-07-23

**Authors:** Eleonora Florio, Simone Rota Biasetti, Ilenia Piazzalunga

**Affiliations:** 1 Psychologist and Psychotherapist, Private Practice, Bergamo, Italy; 2 Department of Human and Social Sciences, University of Bergamo, Bergamo, Italy; 3 Bachelor of Engineering for Health Technologies. Department of Management, Information and Production Engineering, University of Bergamo, Dalmine, Italy; 4 Clinical Psychologist, Private Practice, Bergamo, Italy

**Keywords:** adolescents, family therapy, Linear Mixed-Effects (LME) modeling, NSSI, semantic polarity theory, suicidal ideation, systemic relational therapy, text mining

## Abstract

**Introduction:**

Retracing the theme of what can legitimately be considered “evidence” in psychotherapy, the present work shows the application of an *ad hoc*, rule-based text mining program specifically designed to analyze therapy session transcripts. Developed within a systemic-relational framework, and grounded in Semantic Polarities Theory, the program works through a hybrid human-in-the-loop approach and provides data for subsequent descriptive and inferential analyses on meaning transformation starting from consultation sessions.

**Participants:**

The present study examines the clinical case of an adolescent girl with untreated past non-suicidal self-injury (*N*SSI) and suicidal ideation. Before moving to the joint family work phase, family therapy consultation was conducted in separate sessions within an alternating parent-adolescent format.

**Methods:**

Overall semantic activity was compared across events with Wilcoxon rank-sum tests. In categorical comparisons**,** data were analyzed using chi-square tests, with df-scaled Cramer's *V* to measure effect sizes and Monte Carlo *p*-values. Initial subjects' positioning profile across Family Semantic Grids (FSGs) was described using semantic heatmaps and main polarities use. Linear Mixed-Effects (LME) models were implemented to track longitudinal changes in the subjects' positioning, specifically investigating the therapist's influence.

**Results:**

Results indicated that the therapist introduced a significantly lower proportion of new meanings across all comparisons during the consultation phase. LME models showed significant intensity shifts toward positive-valued meanings, supported by *ICC*, $R_{m}^{2}$ and $R_{c}^{2}$ values, and inform on the significant specific use of Narrated Semantic Polarities (NSP) and Interactive Semantic Polarities (ISP) across participants.

**Discussion:**

Overall, the study supports the feasibility of a semi-automated process for coding and for subsequent analyses of family semantic polarities. In fact, performed analyses demonstrate how therapist positioning and meaning management through re-expression and semantic shifts significantly influenced family members' change in positioning, an effect especially evident for the identified patient. In conclusion, rule-based text mining can yield process indicators that remain clinically legible. Read as “semantography,” results provide a compact, data-grounded map of coupling and early change while staying parsimonious on case disclosure. Limits and future directions of the present pilot study are discussed.

## 1 Introduction

This opening section outlines how the systemic-relational approach in psychotherapy has engaged with the evidence-based movement, a dialogue marked by the attempt to make complexity visible without reducing its meaning—an ongoing challenge shared by several psychotherapeutic traditions. Retracing the theme of what can legitimately be considered “evidence” in psychotherapy, it reflects on the contribution that text-mining methodologies may offer to this matter. The section also provides an overview of Semantic Polarities Theory, whose presentation is essential as it constitutes the theoretical framework that structures and guides the present research.

### 1.1 Beyond protocols: systemic therapy at the crossroads of evidence and complexity

The history of systemic family therapy mirrors the broader trajectory of psychotherapy's encounter with the evidence-based paradigm. When Hazelrigg et al. (1) reviewed outcome studies, they already pointed out that systemic interventions seemed promising but also remarked the limited evidence supporting their effectiveness. Since then, the field has moved forward: in a more recent work adopting a meta-perspective on psychotherapy research methods, Castonguay et al. (2) included systemic therapies among the four main theoretical orientations recognized as evidence-based treatments for a wide range of clinical problems. These approaches often benefit not only identified patients but all members of the family system across diverse conditions—from externalizing and eating disorders to substance use disorders, depression, and psychosis—and have been successfully applied across the life span (children, adolescents, adults) and in multiple settings involving couples, family, individuals, groups, and multifamily interventions (2–9). Moreover, cost-effectiveness analyses suggest that healthcare systems might benefit from integrating systemic therapies into their services (10). In line with this, recent large-scale data from integrated youth mental health programs show that many clients present with moderate to high clinical and psychosocial complexity, pointing to the need for more holistic and multidisciplinary approaches in public healthcare (11).

Yet as Sprenkle (12) argued, the mechanisms of change remain under-explored: although systemic treatments are known to produce effects, relatively few scholars have identified which components of those interventions are the active ingredients and which are inert fillers in specific approaches, so that the “Why” it works remains uncovered. In fact, in a comparative study classifying couples and family therapy models (13), the authors note that not all systemic models share the same level of empirical backing: according to the guidelines of Sexton et al. (14), some models are ranked as reaching only Level I (i.e., showing some empirical support but with methodological limitations), rather than fully evidence-based, that would be a Level III. For instance, the Milan systemic approach (15–18)—well known and widely recognized in Italy and internationally—has been categorized as a level I, therefore not fully evidence-based. Nevertheless, several efforts have been made to reconcile the systemic epistemology with empirical standards. Calls for manualization of systemic family therapy date back more than two decades (19) and the demand for a coherent, integrated assessment battery for outcome variables has persisted in subsequent years (20). Notably, during those years there was a strong impetus to measure systemic therapy and several tools have been validated (21, 22) yet these instruments have not achieved widespread adoption, despite the broader movement toward empirically supported therapies.

The reported reluctance of clinicians to embrace evidence-based practice (EBP) in those years was itself studied by Lilienfeld et al. (23) who proposed a mapping of the underlying sources of resistance and suggested constructive remedies for addressing objections to EBP of the next generation of clinical practitioners and researchers with the iconic opening argument that within psychotherapy training, repeated manifestations of resistance are viewed as cues to investigate the underlying dynamics that sustain them. Other authors have focused instead on the limited effectiveness of training itself, which, despite improving knowledge and attitudes, often fails to produce consistent changes in therapeutic behavior, suggesting that EBP training should be conceived within a systems-contextual framework as a multilevel process (24).

Notably, this issue continues to attract attention; a more recent study with similar focus (25) concluded that clinical professionals generally value the art of psychotherapy more than evidence-based procedures, viewing personal competence as more decisive than adherence to a specific model, while still recognizing therapy as both an art and a science. Indeed, Berg (26) has argued that the tripartite ideal of evidence-based practice remains largely rhetorical. Clinical expertise and patient preferences are still legitimized through scientific validation rather than recognized as distinct and complementary sources of knowledge, revealing a persistent scientocentric bias within the model.

Altogether, the efforts highlighted by several studies (2–9) indicate that the systemic tradition has not resisted the empirical turn but has rather sought to redefine what counts as evidence—developing multilevel frameworks, outcome measures, and manualized procedures that preserve the relational and meaning-oriented essence of its practice. Therefore, one possible explanation for systemic clinicians' reluctance may lie in a tension with standard measurement. This is a tension that can be found in psychotherapy as a whole already at the early 2000s: Westen & Bradley (27) pointed out that both the focus on manuals for discrete disorders and the tendency for judgments of “supported vs. unsupported” treatments risk omitting much of the science and clinical wisdom embedded in practice. Instead, a broader conception of evidence-based practice should integrate multiple evidence sources, accommodate patient complexity beyond categorical diagnoses, and compare new interventions not only with minimal controls but also with existing best practices (27).

Despite growing attention, complexity assessment in clients with mental illness remains an important yet underrecognized area (28). In their recent review Kapustianyk et al. (28) examined the available measures of clinical complexity, showing how these tools could contribute to a better understanding of patients, more accurate referral, and improved coordination between services and levels of care. Although several promising instruments were identified, the authors also note that most still lack direct input from patients themselves—a crucial element for ensuring that any measure can be considered both reliable and truly reflective of the complexity of their experience.

Indeed, more recently psychotherapy as a whole has been argued to require a focus shift from traditional, school-based models toward competence-based orientations that target metatheoretical mechanisms and treatment subgoals, aiming for an evidence-based approach that is finely attuned to the individual's unique nature, context, and problem setting, and doing justice to the complex work of psychotherapy (29, 30). The idea of acephalous instruments was not new: attempts have long been made to develop appropriate measurements maintaining the emphasis on meaning, for instance through the collaborative creation of large datasets across clinicians (31), or later by centering attention on Common Factors (32), or by urging applied psychology to unite around a “common cause” that can foster basic analyses and mid-level constructs capable of being both scientifically progressive and clinically useful (33).

However, to date, these efforts to preserve the richness of meaning within measurable frameworks have not fully resolved the issue. As Kapustianyk et al. (28) note, there remains a need not only to incorporate patients' direct perspectives but also to move toward a shared, evidence-based understanding of what “complexity” actually means. Such an understanding should draw on key notions from complexity theory—including nonlinear dynamics, reciprocal causality, and the delicate balance between order and instability often described as the “edge of chaos”—so that these principles can inform the development and refinement of complexity measures (28). Yet, as we shall see, defining and measuring complexity may itself be a paradoxical endeavor. Indeed, complexity inherently resists conventional measurement, as it challenges both reproducibility and the measurement of general constructs (34), while complex causality undermines the standard assumptions underlying psychological measurement (35). Ultimately, complexity requires models that conceptualize mental disorders as emergent phenomena within dynamic systems—implying nonlinearity, reciprocal causation and systemic interplay—which defy capture by fixed protocols (36).

### 1.2 Text mining between soft data and hard analyses

If complexity resists fixed protocols, it also invites new methodological responses. One promising direction lies in text mining approaches, which seek to capture meaning from clinical language itself, rather than from predetermined variables. In this regard, text mining has emerged as a valuable method for analysing rich textual materials and for giving voice directly to patients—thus avoiding the closed-ended logic typical of self-report questionnaires. Several reviews have underlined its growing use in medical (37, 38) and psychiatric research, where large unstructured corpora can be explored to detect linguistic patterns, meanings, and latent dynamics that would otherwise remain inaccessible, thus representing a powerful method, though not without limitations (39). A recent review on phenomena relevant to adolescent emotion regulation (40) used text mining to expand the scope of the analysis far beyond what would have been possible with a narrative review, substantially increasing the number of studies included and demonstrating how fruitfully this method can be applied. However, its main limitations still concern meaning: the algorithm cannot yet grasp the sense of words as a human interpreter would. Nevertheless, it remains a promising tool for further methodological development (40). A recent proof-of-concept study (41, 42) showed that Multi-Level Models (MLMs) integrated with text-mining techniques can enrich Therapist-Client Process Research (TCPR), providing new analytic opportunities for the study of therapeutic discourse. In a subsequent review of 318 studies (41, 42), the authors identified four methods commonly used in process research—Innovative Moments, Narrative Process, Assimilation of Problematic Experiences, and Conversation Analysis—noting that the first three showed sufficient reliability for partial automation, while content and grammatical features appear suitable for rule-based coding.

The relevance of these methods is further illustrated by their use in internet-based Cognitive-Behavioural Therapy (iCBT), a field that has long provided benchmarks for evidence-based protocols: Mylläri et al. (43) demonstrated that topic modelling through text-mining data offers a feasible and informative approach for examining therapeutic exchanges in iCBT. Likewise, Atzil-Slonim et al. (44) found that topic modelling can extract semantically coherent themes linked to clients' functioning and rupture episodes, with shifts in these topics corresponding to changes in treatment outcome. In a similar vein, Wiegersma et al. (45) designed a Dutch multi-class text-classification model to facilitate screening for several mental disorders and to enhance the accuracy of clinical referrals. Drawing on textual responses provided by 5′863 patients to a standard intake questionnaire, the authors trained a seven-category classifier able to differentiate among anxiety, panic, post-traumatic stress, mood, eating, substance-use, and somatic-symptom disorders, employing a linear support vector machine optimized through nested cross-validation.

The potential of these tools has been quickly recognised and put to work, leading to large-scale reviews that apply intelligent data-mining analyses to enormous datasets—up to 11.3 million scientific publications (46). Moreover, a procedure originally developed in brand management, Emotional Text Mining (47), has been successfully adapted to investigate the cultural representations and symbolic dimensions of psychologists working in assisted reproductive treatment (48). This confirms that the method is methodologically robust, applicable across disciplinary boundaries, and compatible with different analytic frameworks, provided that methodological choices are tailored to the specific area of inquiry. The value of text-based analyses is increasingly recognized: when applied with methodological care, these methods can capture and organize large textual datasets with a precision that allows for systematic synthesis across studies. For example, Li (49) analyzed 564 papers on the use of chatbots in psychological counselling, illustrating how such methods can handle large corpora efficiently. Therefore, the rapid expansion of text mining approaches suggests that they may soon be increasingly employed in large-scale syntheses—such as meta-reviews or mega-analyses like the recent comprehensive work of Carr et al. (50)—so that computational tools can assist, rather than substitute, the human effort still required for evidence aggregation.

Yet, after acknowledging the potential of text mining approaches, it seems worth reconsidering how psychotherapy research might preserve meaning rather than sacrifice it. In fact, meaning itself can be the starting point of analysis, and new methodologies may allow us to preserve more of the complexity of clinical phenomena within reach rather than reducing it. Such complexity tends to remain case-bound because, as Taborsky (51) observed, searching for general laws of complexity may be misguided, since what emerges in one domain does not necessarily illuminate another. From this perspective, the study of a single therapeutic case may already provide a legitimate entry point for understanding that specific psychological constellation, refining tools that can later be applied to other cases, thereby allowing different forms of complexity to be related without losing their specificity. Following Eronen's (35) argument, such an approach aligns with a view of psychological measurement as a form of “soft” measurement: distinct from the “hard” models of the physical sciences, yet capable of generating valid and useful knowledge about the dynamic, causally entangled systems that constitute human experience. This, however, does not preclude the use of hard analyses on soft data—as already exemplified by Smink et al. (41, 42)—an integration likely to represent the most fruitful way forward in psychotherapy research field.

In this sense, epistemically justified inferences in psychology require methods able to analyze individuals' unrestricted verbal responses, rather than standardizing them into pre-defined constructs (52). Nonetheless, as Uher (52) warns, the increasing use of AI-based language models to generate fixed descriptions of psychological phenomena risks perpetuating the discipline's core epistemic error, which is the loss of meaning in the process of quantification. By contrast, rule-based text analysis represents a meaningful form of automation—using computational procedures to extend, rather than replace, human understanding—since, unlike example-based machine-learning models trained on large, annotated datasets, it relies on expert-defined linguistic rules that ensure transparency and interpretive control in exploring clinical discourse (41, 42). While developed within the broader domain of computational social sciences, hybrid human-machine workflows have been found to increase efficiency and accuracy through iterative human validation (53). Therefore, in the present study, a rule-based approach is adopted within a specific theoretical framework as an automation-assisted, clinician-validated procedure, aimed at testing the potential of text mining to capture meaning without losing complexity in a large corpus of textual data.

### 1.3 The conversational roots of meaning: outlining semantic polarities theory

The theoretical reference of this study is Semantic Polarities Theory (54–56), which stems from the Milan approach (15–18) and redefines the traditional systemic orientation in cognitive-constructivist terms. Since the present research is based on clinical material from cases conducted by the first author within Ugazio's psychotherapeutic model, it seems useful to provide a brief outline of Semantic Polarities Theory and the key theoretical background behind it.

Ugazio's elaboration (54–56) stems from cognitive premises derived from Kelly's Personal Construct (57), discourse on how systems of meaning can hold inner contradictions and remain coherent (58–60), and Guidano's framework (61), in which psychopathology is conceived as a science of meaning (62). Though, a decisive epistemological shift led to redefine such cognitive foundations within a constructionist framework, where bipolar constructs, cognitive conflict, and personal meaning are seen as grounded in the relational and narrative matrix of human experience. A central point concerns the irreducibility of triadic configurations (55, 56, 63, 64): meaning does not emerge from inner structures of a closed individual system, nor from the linear interaction of two minds, but from the reciprocal positioning of at least three participants within a relational field. In this framework, family is conceived as a privileged context of meaning organization, and individual patterns are understood as participation in shared family semantics representing systems of shared oppositions that are collectively constructed, emotionally charged, and permeated by cultural influence.

It is precisely this widening of perspective—from the individual mind to the conversational family system—that integrates the legacy of cognitive authors with social constructionism within the epistemology of Gregory Bateson (65–68), whose systemic vision becomes a cornerstone of the Semantic Polarities model, along with Positioning Theory (69–71), where the Self is conceived as the position one adopts in ongoing conversations with others. Within Semantic Polarities Theory, it is assumed that a shared family semantics with salient meanings organized into opposing polarities, is the context in which each member participates in conversation, interacts, and constructs reality in ways that may differ or even conflict with those of others. Therefore, family members necessarily position themselves and, by co-positioning others, anchor interdependent identities.

In this framework, Ugazio's central thesis is that the personality organizations underlying the four major groups of phobic, obsessive-compulsive, eating/feeding, and depressive disorders, correspond to as many family semantics: respectively, the semantics of freedom, goodness, power, and belonging. These dominant meanings, though necessary, are not sufficient for the psychopathological onset, for which what proves crucial is the specific position each person assumes along the salient family polarities. Thus, symptoms arise when a person is deprived of a coherent narrative frame within which to define the Self, with subsequent impossibility to integrate one's lived experience and personal meanings within the available or compatible positions. The onset of disorder therefore gives rise to a psychopathological configuration that resolves this impasse in a paradoxical way, opening a new conversational position, through the symptom. Such new position “(dys-)functions” because it escapes the traditional allocation of values and unsettles the order of meanings existing in the original context—at the cost of constraints and suffering.

### 1.4 Aims and objectives

The present study explores how text-mining techniques can be applied within the framework of Semantic Polarities Theory to support an evidence-based understanding of meaning reorganization in systemic-relational psychotherapy. Building on previous developments of the Family Semantic Grid [FSG; (72–75)], the study examines whether rule-based computational procedures can assist clinicians in identifying recurrent semantic polarities within therapeutic discourse and process, while preserving interpretive depth. Accordingly, this pilot project aims to:

(a) Develop and test a preliminary procedure for the partial automation of the Family Semantic Grid using rule-based text-mining techniques for coding procedures;
(b) Analyze meanings identified and codified through the program, in order to explore how semantic polarities co-occur, shift, and reorganize across therapeutic dialogues, with a specific focus on the initial phases of consultation, which is critical for the construction of the therapeutic alliance and for a generative and constructive start of the therapeutic process;
(c) Explore the conversational patterns across sessions, mapping conversational markers of process-level effectiveness and offering a replicable framework for future studies on systemic meaning construction and therapy conversations analyses.

This first application is conceived as a feasibility step toward the creation of a digital environment for mapping and visualizing the organization of family meanings—a process that, in later stages, may evolve into more complex forms of graphical representation of conversational meaning patterns derived from clinical narratives.

## 2 Materials and methods

### 2.1 Participants and procedure

In order to address the aims of the present study and to situate the application of text-mining procedures grounded in a rule-based approach within a clinically meaningful context, a single clinical case conducted by the first author was selected for analysis. In line with the study's focus, limited contextual and historical information about participants is reported and all identifying characteristics were anonymized, while preserving clinical isomorphism (any resemblance to actual persons, events, or locales is purely coincidental). The case was managed in accordance with the Italian Code of Ethics for Psychologists (76).

The case was conducted by the first author, in her private practice based in Bergamo and concerns a 14-year-old girl, hereinafter referred to with the fictitious name “Irene,” presenting with suicidal ideation and episodes of non-suicidal self-injury (NSSI) described as cutting behaviors reported to have occurred during the previous year and not present at the time parents contacted the therapist. During the first session, it was observed that the adolescent presented with a small burn on her hand, which was reported as accidental. The first contact with the therapist was triggered by the disclosure of suicidal ideation (March 2023); the girl was attending the final year of lower secondary school (middle school) and was therefore approaching the transition to upper secondary education. Irene had received a diagnosis of epilepsy at the age of eight and a diagnosis of Attention-Deficit/Hyperactivity Disorder (ADHD) at the age of 10—a relatively common comorbidity (77, 78),—therefore she had been consistently followed over time by different treating psychologists and child neuropsychiatrists. Moreover, Irene willingly attended individual sessions with the school psychologist with whom she reported a positive relationship and to whom she disclosed the suicidal ideation that led to the referral.

The parents had been separated since Irene was seven years old, and their co-parental relationship was and remains highly conflictual, with occasional periods of improved collaboration. Irene lived primarily with her mother (47 years old) and maintained regular contact with her father (50 years old), which was less frequent due to post-separation caregiving arrangements. Both parents had a university-level education and a good level of familiarity with healthcare contexts; at professional level it is sufficient to highlight that both had very demanding commitments.

The therapist conducted two separate preliminary phone interviews with each parent in order to collect their perspectives and to introduce the systemic-relational framework (i.e., recordings, one-way mirror, etc). Informed consent was obtained for both the psychological consultation and any subsequent treatment, with detailed information provided regarding the clinical setting. At a later stage of the therapeutic process, not included in the present work, the parents provided additional informed consent for the use of all clinical material for research purposes and for its inclusion in scientific publications.

During the consultation phase, which constitutes the focus of the present study, standard clinical tools were used to frame the case, starting with the Telephone Form (79), used to structure the initial phone contact and to collect basic information relevant for setting definition, symptoms exploration, and preliminary hypotheses about individual and family functioning. Given the high level of parental conflict, it was proposed to begin with a consultation phase structured through alternating sessions, in which Irene attended meetings together with her father and, in separate sessions, together with her mother, proceeding in parallel. Both parents agreed to this arrangement and were available and collaborative in their interactions with the therapist, as the alternating format was experienced by both as more manageable given the level of conflict. The other clinical tools used were the Temporal Table (80), employed to record and integrate the different timelines explored during the consultation—particularly relevant in this case given the alternating work with each parent and the need to cross-reference information emerging from separate sessions—and the Genogram (81–83), constructed in the early sessions to represent family structure, relational patterns, and significant transgenerational elements.

Parents' initial request was primarily focused on the wish for their daughter to regain serenity and overall emotional well-being, since the psychological consultation was prompted by the disclosure of suicidal ideation, but parental explicit concern appeared initially moderate. Despite this, both parents were collaborative and available in their interactions with the therapist and made significant organizational efforts to attend the sessions, for example, the father regularly traveled from the Monza area to Bergamo (about 40 km distance), involving substantial time and logistical demands.

Consistently with a systemic perspective, the therapist's speech was coded and examined in the same way as the other speakers, with the only analytic difference of specify therapist positioning as the predictor in the LME models, reflecting the study's theoretical focus.

### 2.2 Instrument: program development and structure

To analyze the transcripts of the therapeutic sessions, a custom program written in the Python programming language (84) was developed for this purpose using a rule-based text-mining approach grounded in the Family Semantic Grid instrument [FSG; (72–75)], rooted in the systemic-relational tradition; for a detailed theoretical and clinical presentation of the FSG, the reader is referred to these original contributions. The sessions were manually transcribed by the third author of the present study without the use of automated transcription tools, and non-verbal expressions visible in the video recordings were included.

The program, developed from scratch without relying on pre-existing packages, was designed to support the partial automation of FSG analytic procedure, while preserving the central role of the clinician in the identification, interpretation, and evaluation of meanings. In this sense, it implements a human-in-the-loop procedure (85, 86) in which clinical expertise explicitly guides the analytic process, rather than supervising autonomous algorithmic decisions, combining interpretive depth with systematic coding support, workflow efficiency, and structured, traceable data management. In operational terms, the human-in-the-loop component requires the clinician to associate the words prompted by the program with FSG meanings, or to identify and associate additional elements not automatically detected, while the program provides an optimized coding environment designed to support the organization of the coded material.

The software relies on a lexical library enriched with synonyms traceable to the original lexical items of the FSGs; the initial version of the library included 1,259 items. The program identifies in a transcript the lexical items associated with the library's records, applying strict suffix-stripping procedures to recognize the words' root despite natural linguistic variations. This may increase the likelihood of false positives rather than missed words, but these can be easily skipped by the clinician. The possibility to add lexical items proved particularly useful for refining the case-specific vocabulary, including metaphorical expressions and context-bound interactions, allowing the same meanings to be consistently identified across sessions; at the end of the coding process, the library had increased by 173 items, which could be detected by the program through the same suffix-stripping procedure applied to the initial library. Moreover, for each record, a continuum ranging from −4 to +4 was implemented to allow the coding of polarity intensity and value (positive, neutral, and negative) in line with the continuum-based procedure described within the FSG approach. The choice of a 4-step range on each side of the continuum was inspired by methodological reasoning commonly adopted in the construction of Likert-type response formats, particularly with regard to limiting the overuse of intermediate categories that may absorb coder uncertainty or ambivalence (87–89). Intermediate positions were treated as graded deviations toward one pole or the other, providing a centered formal representation of the continuum for the median position. Specific positioning was not encoded through a single dedicated parameter, since it can be inferred integrating each polarity label with its intensity and value orientation, thus maximizing coding flexibility. Similarly, the explicit indication of semantic polarities as open or closed (72) is no longer required at the coding stage, as these features can subsequently be identified during data analysis if needed. Accordingly, distinctions such as first expressions, re-expressions, and semantic shifts (72) were not coded, since such semantic status could be directly reconstructed from the ordered sequence of coded meanings: polarity occurrences were ordered by their progressive index, reflecting their order of appearance in the dialogue or interaction, and linked to the immediately preceding occurrence of the same polarity. In addition, for each narrative or interactive semantic polarity expressed, the program prompts the therapist to specify both the referent to whom the meaning applies and the speaker producing it.

In the present study, the segmentation into narrative units (72) was not introduced, since entire sessions were analyzed in their full temporal sequence, with conversational turns treated as the primary unit of analysis, in line with a microanalytic sequential approach to psychotherapy process research (90). This choice was motivated by the aim of examining how meanings co-occur, persist, and reorganize across dialogue as it unfolds turn by turn.

### 2.3 Clinical material

Events selected for the analysis were drawn from the initial phases (consultation) of the therapeutic process. The corpus included the initial separate phone contacts with the father and the mother, the first session with the mother and Irene, and the first session with the father and Irene. Such events provide a snapshot of the initial functioning and organization of meanings at the outset of the process.

The therapeutic phase formally began with the provision of therapeutic feedback and included the third session with the father and Irene and the third session with the mother and Irene. In addition, part of the material from session 12 was included, since this represented the first session in which an important objective was achieved: making a joint family session possible, having both parents and Irene present together in the therapeutic setting. For this latter session, only the central third was analyzed, since semantic profile should remain unchanged (91): it was included to capture a representative segment of the first joint family session following a consolidated phase of separate work with Irene and each parent, a key objective of the therapeutic process in this case. Table 1 provides an overview of the coded clinical material; sessions were scheduled with a minimum interval of two weeks and sometimes longer, to accommodate parents' organizational constraints. Given Irene's greater involvement in the in-person work, the timing of sessions was calibrated to avoid excessive burden.

**Table 1:** Overview of the coded clinical material.

**ID**	**Setting**	**Participants**	**Phase**	**Description**	**Timing**	**IRR (Cohen's *κ*)**
P1	Phone call	Mother	Consultation	Initial contact	07/03/23	.62
P2	Phone call	Father	Consultation	Initial contact	07/03/23	.66
S1	Session	Mother and Irene	Consultation	First session	13/03/23	.72
S2	Session	Father and Irene	Consultation	First session	15/03/23	.66
S5	Session	Father and Irene	Feedback session	Third session with Father	14/04/23	.69
S6	Session	Mother and Irene	Feedback session	Third session with Mother	21/04/23	.64
S12^a^	Session	Mother, Father & Irene	Therapy	First joint family session	05/08/23	.67

*Note*. Session: in-person session.

a For session S12, only the central third of the session was included for follow-up purposes.

IRR, Inter-Rater Reliability.

Occasional phone contacts with one parent were allowed upon request, mainly to support the therapeutic alliance and address concerns not suitable for the sessions with Irene, who understood them as part of a shared adult responsibility supporting the therapeutic work, while keeping her protected and central to the process. These contacts were transparently acknowledged and balanced by comparable availability to the other parent.

### 2.4 Data analysis

Analyses on the data of the consultation phase, both descriptive and inferential, were performed in R (Version 2026.01.0 + 392) and included distribution summaries of polarity value and intensity both for NSP and ISP, presented by speaker and event, with percentages of semantic-status types (expressions, re-expressions, and semantic shifts). Group differences on continuous variables were tested using Wilcoxon rank-sum tests. Chi-squared test of independence was performed to assess significance across contingency tables; distribution of clinical data may easily appear sparse (i.e., rare but potentially meaningful occurences), and *χ*^2^ asymptotic assumptions could be violated; therefore, whenever low counts were expected, *p*-values were estimated via Monte Carlo simulation—already implemented in “chisq.test” package (92)—which provides more reliable inference under sparsity. Cramer's *V* was calculated as effect size index for each *χ*^2^-test, and threshold values were interpreted using Cohen's (93) framework with *w* conventional benchmarks of .10, .30, and .50 (i.e., small, medium, and large), though adapted to the effective degrees of freedom (df_eff_) of each specific table (cf. Table 2). This procedure is based on how Cohen (93) defined the relationship between the chi-square effect size index *w* and the generalized association coefficient *ϕ*′ (equivalent to Cramer's *V*) as:

$$V=\phi^{\prime }=\sqrt{\frac{\chi^{2}}{N\cdot df_{eff}}}$$

where df_eff_ = min(r − 1, c − 1) for an r·c contingency table. Using Cohen's conventional benchmarks for *w* (.10, .30, .50), df-scaled reference values for *V* follow from:

$$V=\frac{w}{\sqrt{df_{\mathrm{eff}}}}.$$

**Table 2:** Reference thresholds for Cramer's *V* as a function of effective degrees of freedom (df).

df*_eff_*	Small *V*	Medium *V*	Large *V*
1	.10	.30	.50
2	.07	.21	.35
3	.06	.17	.29
4	.05	.15	.25
5	.04	.13	.22
6	.04	.12	.20
7	.04	.11	.19
8	.04	.11	.18
9	.03	.10	.17
10	.03	.09	.16
11	.03	.09	.15
12	.03	.09	.14
13	.03	.08	.14
14	.03	.08	.13
15	.03	.08	.13
16	.03	.08	.13
17	.02	.07	.12
18	.02	.07	.12
19	.02	.07	.11
20	.02	.07	.11
21	.02	.07	.11
22	.02	.06	.11
23	.02	.06	.10
24	.02	.06	.10
25	.02	.06	.10

*Note*. df, Effective df [min(r−1, c−1)].

The table reports thresholds up to 25 df to match the maximum df in this study (22) and complete the series.

For this reason, in the present work df-scaled reference values for Cramer's *V* derived from Cohen's benchmarks are considered; the following Table 2 shows the calculated thresholds:

Inter-Rater Reliability (IRR) was assessed through Cohen's *κ* (94) by comparing a subset of the primary coding with the corresponding subset independently coded by an additional rater. The main coder was the therapist who conducted the sessions (i.e., first author of the present study). The additional rater, who worked only on the middle 33% of each transcript for IRR purposes, was the third author of the study and had previously transcribed the sessions, thus being familiar with the clinical material. No specific training was conducted, except for the instruction to study the original publications presenting the validation and use of FSG and for an explanation of the program's coding characteristics. The two coders worked independently, without discussing coding decisions, and followed the presented coding rules (further specified in Supplementary Material). The agreement for this measure was *κ* = .67, indicating a substantial level of agreement according to the guidelines (95). Cohen's *κ* was calculated by the second author of this study, rather than by the coders themselves; this author was responsible for the program development and part of the data analysis, working only on coded and anonymized material, without direct access to the clinical transcripts.

Concerning data preparation for the analysis, we linearly rescaled intensity scores of the median pole (neutral) to a −1 to +1 continuum, so that mid-range (near-zero) variations remain visible and interpretable rather than being flattened. The semantic status variable was created by coding occurrences as “Expression” when they constituted the first instance of that polarity overall, or when the previous instance occurred in an event in which the current speaker was absent. “Re-expression” was coded when a prior valid occurrence with the same value direction (positive vs. negative) was available, allowing intensity changes. “Semantic shift” was assigned when the polarity was reintroduced with a reversal in value's sign. While Ugazio et al. (72) conceive semantic shifts also as a movement across the four grids (Belonging, Freedom, Goodness, Power), here shifts were operationalized conservatively as direction reversals; semantic domains that carry the main dynamics of re-elaboration will be visible from subsequent analysis.

Family members' positioning (i.e, polarity intensity and value) was modeled as a function of therapist's positioning using Linear Mixed-Effects models (LME) implemented in MATLAB (fitlme). This analytical choice was made to account for the nested structure of the data (i.e., speakers nested within different events). All valid data was retained since each person's verbal intensity value was considered as the result of multiple underlying, approximately independent factors (e.g., emotional state, cognitive load, therapeutic context, etc.), each contributing additively to the observed intensity. Let $X_{1},X_{2},\ldots ,X_{n}$ represent these factors with finite mean $\mu_{i}$ and variance $\sigma_{i}^{2}$. By the Lindeberg-Feller form of the Central Limit Theorem (CLT), the sum of these factors converges to a standard normal distribution even if the $X_{i}$ are not identically distributed, provided no single factor dominates the variance (96):

$$Z_{n}=\frac{\sum_{i=1}^{n}(X_{i}-\mu_{i})}{\sqrt{\sum_{i=1}^{n}\sigma_{i}^{2}}}∼N(0,1),\;n\to \infty$$

Values of the observed intensity range between −4 and +4 on the rating scale, under the CLT framework, can be treated as approximately normally distributed, justifying the inclusion of all data points in subsequent LME (97). In the context of the present study, extreme values are retained as potential clinically meaningful material, such as—for example—local maxima (98). Each LME model includes the person's prior positioning (lag of one turn) as a fixed-effect predictor to account for short-term temporal autocorrelation. The appropriate temporal matching window between therapist and other speakers' turns was individuated through a sensitivity analysis, evaluating the more stable range of effect through measures of standardized coefficients and effect size. Thereby empirical stability was used to separate noise from the interactional flow worth to be modelled, balancing robustness with sufficient data inclusion. A conservative window of ±7 semantically coherent conversational turns (i.e., those associated with the same polarity) was selected as representative. The same window was applied uniformly across all participants, in order to ensure methodological consistency and comparability of estimates across subjects. Therefore, each positioning expressed or enacted by family members was aligned with therapist's, using a polarity-constrained matching (within ±7 turns), which proceeds sequentially through conversation. This procedure constructs a therapist-centric table: other speakers' positioning values are assigned to adjacent therapist's turns, allowing the model to assess how their responses evolve longitudinally in relation to therapist's meanings management on the same polarity. This approach focuses the analysis on how family members' expressions change in relation to therapist input, rather than the converse.

LME models were therefore based on the relevant hypotheses with fixed effects including therapist's positioning (controlling for the number of days between events), with the random intercept for events (phone call or session) included in all models to account for the clustering of observations within them. Following the procedure of Florio et al. (99), Intraclass Correlation Coefficients (*ICC*s) from intercept-only (null) models were computed to motivate the multilevel analysis. *ICC*s were interpreted using conventional benchmarks (100)—poor (< .05), small-to-moderate (from .05 to .75), good (from .75 to .90), and excellent (> .90)—while acknowledging that values in the .05–.25 range are common in social-science applications (101). If *ICC* = 0, indicating no variance attributable to the random-intercept, we retained the model whenever the fixed-effect estimate was statistically significant, in order to preserve comparability across models. Finally, we complemented each LME with marginal ($R_{m}^{2}$) and conditional ($R_{c}^{2}$) variance explained respectively by fixed effects alone vs. the full model, thus informing on predictor's effect size (102–104).

## 3 Results

This section reports results from analyses of data coded through the program developed for this study. We first describe the therapy initial phase and then examine the LME model that also covers the feedback sessions and the last session (S12—only central third coded), which is the first session attended by all family members.

### 3.1 Descriptives of the initial phase

Table 3 summarizes the semantic orientation expressed by each speaker during the first phone call and the first session, distinguishing between narrated (NSP) and interactive semantic polarities (ISP) along the value axis (ranging from −4 to +4).

**Table 3:** Semantic orientation (−4 to +4) and occurrence of narrated and interactive semantic polarities during early interactional events, with Wilcoxon-based comparisons.

Speaker	Event	FSG type	*N*	*M*	*SD*	*sk*	*ku*	*CI* 95%		*P* (FP vs. FS)	*R* (FP vs. FS)	*P* (NSP vs. ISP)	*r* (NSP vs. ISP)
								*low*	*high*				
M	FP	ISP	11	1.91	1.76	−2.532	7.127	0.728	3.092	-	-	.002**	.386
		NSP	55	−0.65	2.37	0.274	−1.600	−1.291	−0.009	.573	.059		
	FS	ISP	24	0.38	2.34	−0.697	−0.838	−0.608	1.368	.033*	.364	.373	.113
		NSP	39	−0.36	2.54	0.111	−1.741	−1.183	0.463	-	-		
F	FP	ISP	50	2.08	1.48	−0.613	−0.948	1.659	2.501	-	-	.000***	.382
		NSP	111	−0.68	3.10	0.390	−1.600	−1.263	−0.097	.972	.002		
	FS	ISP	143	1.52	2.19	−1.202	0.418	1.158	1.882	.198	.093	.000***	.271
		NSP	123	−0.51	3.23	0.232	−1.704	−1.087	0.067	-	-		
I	FS°	ISP	236	1.80	2.31	−1.333	0.514	1.504	2.096	-	-	.000***	.404
		NSP	333	−0.88	3.01	0.480	−1.469	−1.204	−0.556	-	-		
T	FP°	ISP	126	1.67	1.93	−1.088	0.832	1.330	2.010	-	-	.045*	.167
		NSP	20	0.65	2.32	−0.170	−1.361	−0.436	1.736	.514	.055		
	FS°	ISP	427	1.61	1.60	0.096	−1.470	1.458	1.762	.321	.042	.000***	.155
		NSP	120	0.91	1.84	0.047	−0.463	0.577	1.243	-	-		

*Note*. ^*^p < .05.

** *p* < .01.

*** *p* < .001.

*p* values refer to Wilcoxon rank-sum tests; effect sizes are reported as *r* (*Z*/√*N*).

Speaker = M (Mother), F (Father), I (Irene), T (Therapist).

FP, First Phone call.

FS, First in-person Session.

FP°, First Phone calls (P1 + P2).

FS°, First Sessions (S1 + S2).

ISP, Interactive Semantic Polarities.

NSP, Narrated Semantic Polarities.

*Sk*, Skewness.

*Ku*, Kurtosis.

-, repetition or N/A.

Across speakers and interactional events, interactive semantic polarities (ISP) generally showed higher (i.e., more positive) semantic orientation scores, whereas narrated semantic polarities (NSP) were generally characterized by lower or negative orientations. Irene (I) showed the strongest contrast in positioning between NSP and ISP, as well as greater dispersion of NSPs (less homogeneous values). The therapist displayed instead a predominantly positive tone. Concerning the mother (M), the separation between polarity type (NSP/ISP) appears clearer in the first phone contact. Finally, several distributions show marked asymmetry and/or peakedness, which supports the use of rank-based comparisons.

For both parents and the therapist, contrast between NSP and ISP emerged more clearly than differences across the two events examined, with no significant change in semantic orientation between the first phone call and the first session in most conditions, other than what was observed for the mother, who showed a significant difference with a medium effect size, consistent with a markedly weaker positive orientation of ISP when she was in session together with her daughter, compared to the phone contact where she was speaking alone with the therapist.

**Figure 1:**
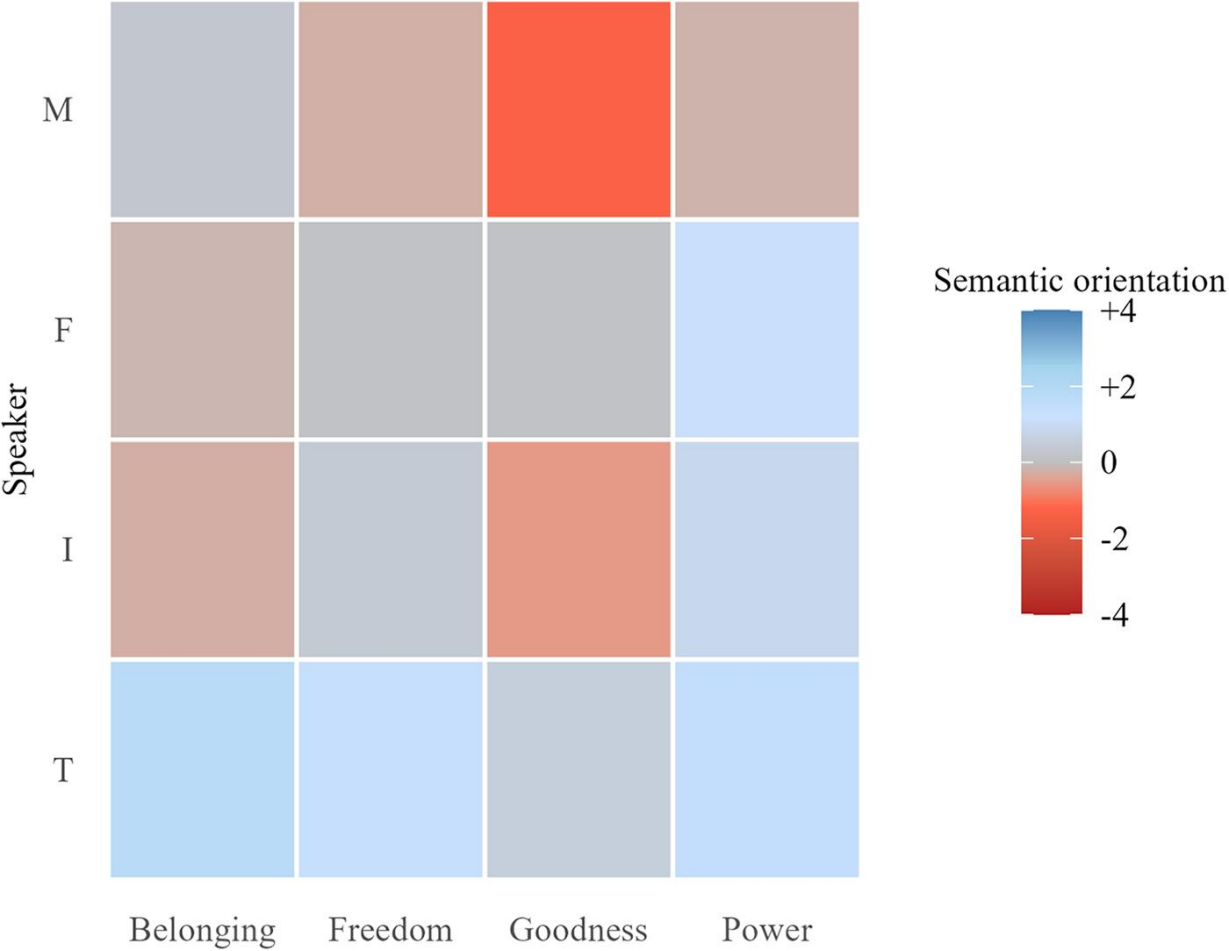
Heatmap: global semantic map (P1, P2, S1, S2): mean semantic orientation (−4 to +4) for each speaker and grid, computed from signed intensity and averaged across polarity occurrences. *Note*. Speaker: I, Irene; F, father; M, mother; T, Therapist. FSG semantics in alphabetical order: Belonging, Freedom, Goodness, Power.

To complement these contrasts regarding within-speaker semantic activity, a global view of the initial semantic structure is provided, aggregating narrated and interactive polarities within each semantic grid. To this purpose, Figure 1 displays a heatmap summarizing the initial semantic structure across the four FSG semantics—Belonging, Freedom, Goodness, and Power—during the beginning of the consultation phase (P1, P2, S1, and S2). For each speaker and grid, semantic orientation was computed by integrating polarity valence (always according to the speaker), intensity and averaging signed intensity values across all occurrences, thus providing a single global index per cell.

The heatmap highlights a positive orientation for the therapist across all grids, with mostly consistent values rather than grid-specific shifts, with the meanings of Goodness closest to a neutral valence. In contrast, family members display more grid-specific profiles, with Irene presenting the most split pattern, with negative meanings in Belonging and Goodness, contrasted with positive orientation of meanings in Freedom and Power.

While these results describe the semantic orientation and grid at the global level, they do not yet distinguish between each speaker's contributions in terms of meanings newly introduced vs. meanings taken up and re-elaborated within the dialogue or interaction. To address this issue, the sequential emergence of semantic polarities during the events of the initial phase was examined, distinguishing semantic status type (expressions, re-expression, semantic shifts). Table 4 shows these results across speakers and event.

**Table 4:** Semantic status: expressions, re-expressions, and semantic shifts across initial interactional events.

**Speaker**	**Event**	**FSG type**	**Semantic status**	***N***	**% within same speaker**	**% of entire event**
M	P1	ISP	Expression	5	7.6	4.6
			Re-expression	6	9.1	5.5
			Semantic shift	1	1.5	0.9
		NSP	Expression	34	51.5	31.2
			Re-expression	15	22.7	13.8
			Semantic shift	5	7.6	4.6
	S1	ISP	Expression	7	11.1	1.6
			Re-expression	9	14.3	2.1
			Semantic shift	8	12.7	1.8
		NSP	Expression	12	19.0	2.8
			Re-expression	16	25.4	3.7
			Semantic shift	11	17.5	2.5
F	P2	ISP	Expression	15	9.3	5.7
			Re-expression	24	14.9	9.1
			Semantic shift	11	6.8	4.2
		NSP	Expression	71	44.1	26.9
			Re-expression	27	16.8	10.2
			Semantic shift	13	8.1	4.9
	S2	ISP	Expression	20	7.5	2.0
			Re-expression	82	30.8	8.1
			Semantic shift	40	15.0	4.0
		NSP	Expression	45	16.9	4.5
			Re-expression	47	17.7	4.7
			Semantic shift	32	12.0	3.2
I	S1	ISP	Expression	20	9.3	4.6
			Re-expression	36	16.7	8.3
			Semantic shift	14	6.5	3.2
		NSP	Expression	71	33.0	16.3
			Re-expression	46	21.4	10.6
			Semantic shift	28	13.0	6.4
	S2	ISP	Expression	21	5.9	2.1
			Re-expression	99	28.0	9.8
			Semantic shift	45	12.7	4.5
		NSP	Expression	64	18.1	6.3
			Re-expression	82	23.2	8.1
			Semantic shift	43	12.1	4.3
T	P1	ISP	Expression	12	27.9	11.0
			Re-expression	23	53.5	21.1
			Semantic shift	0	-	-
		NSP	Expression	7	16.3	6.4
			Re-expression	0	-	-
			Semantic shift	1	2.3	0.9
	P2	ISP	Expression	19	18.4	7.2
			Re-expression	44	42.7	16.7
			Semantic shift	28	27.2	10.6
		NSP	Expression	8	7.8	3.0
			Re-expression	3	2.9	1.1
			Semantic shift	1	1.0	0.4
	S1	ISP	Expression	20	12.7	4.6
			Re-expression	45	28.7	10.3
			Semantic shift	45	28.7	10.3
		NSP	Expression	16	10.2	3.7
			Re-expression	10	6.4	2.3
			Semantic shift	21	13.4	4.8
	S2	ISP	Expression	22	5.6	2.2
			Re-expression	190	48.7	18.8
			Semantic shift	105	26.9	10.4
		NSP	Expression	25	6.4	2.5
			Re-expression	21	5.4	2.1
			Semantic shift	27	6.9	2.7

*Note*. Speaker: I, Irene; F, father; M, mother; T,  Therapist.

NSP, Narrated Semantic Polarities.

ISP, Interactive Semantic Polarities.

Expression, meaning newly introduced in the dialogue.

Re-expression, meaning taken up from previous turns, allowing for change in intensity.

Semantic shift, meanings that undergo an overturning of their value orientation (–/n/+).

As can be seen in Table 4, from the first phone contact (P1) to the first in-person session (S1), the mother reduced narrated first expressions and remained active in re-expressions with NSP re-expressions becoming proportionally more prominent, and ISP semantic shifts increasing in the first session with the daughter. First phone call with the father (P2) was characterized by a substantial proportion of NSP first expressions, which consequently decreased in the first in-person session with Irene (S2). In such event, father's activity concerning ISP increased, with higher levels of semantic activity—if compared with the mother—and being more strongly organized around re-expressions and semantic shifts. Irene showed a clearly different semantic activity across the two in-person sessions. If compared with S1 (first session with the mother), Irene's first expressions in S2 (with the father) seemed to decrease, while meanings were handled more through ISP re-expressions and semantic shifts. Still in S2, therapist's activity was also more strongly concentrated on ISP re-expressions (and semantic shifts) in the interaction activity with the father and Irene.

To assess whether the therapist's semantic status contribution differed from that of each interlocutor, chi-square tests were conducted comparing the therapist separately with the mother, the father, and Irene, alongside with Cramer's *V* for effect sizes: the therapist showed a lower proportion of first expressions across all comparisons with the magnitude of this difference being intermediate in relation to the mother [*χ*^2^(2) = 43.5, *p* < .0001, *V* = .23] and smaller with Irene [*χ*^2^(2) = 31.1, *p* = .0001, *V* = .16] and the father [*χ*^2^(2) = 41.9, *p* < .0001, *V* = .19].

During the two phone contacts (P1 and P2), a significant association emerged, *χ*^2^(2) = 20.4, *p* < 0.0001, *V* = .37 (large effect), indicating that ISP were predominantly handled by the therapist through re-expressions and semantic shifts and the same association was observed when aggregating the first in-person sessions (S1 and S2): *χ*^2^(2) = 53.6, *p* < .0001, *V* = .31 (medium effect).

**Figure 2:**
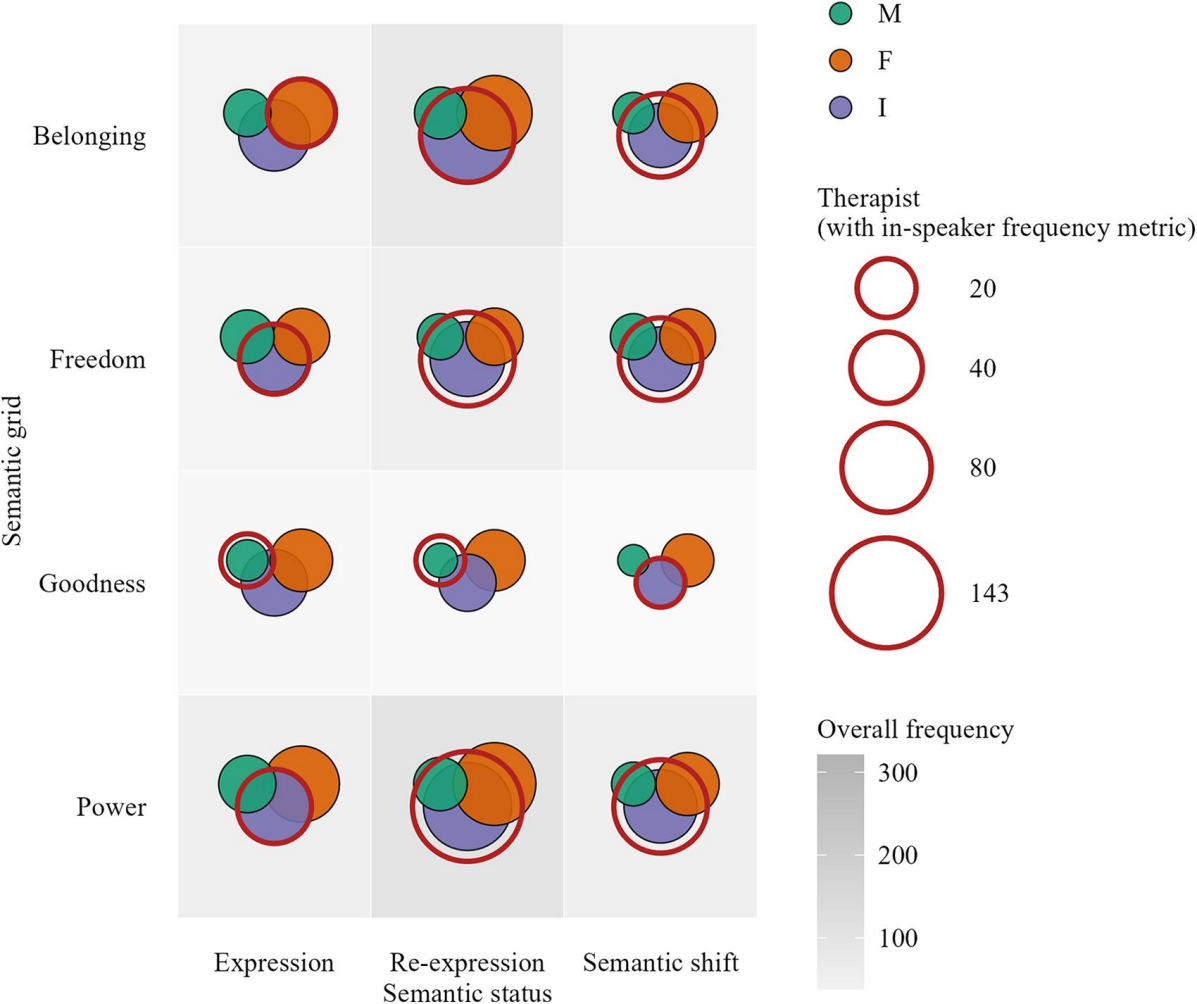
Semantic activity across grids and speakers (initial phase: P1, P2, S1, and S2). *Note*. Speaker: I, Irene; F, father; M, mother. FSG semantics in alphabetical order: Belonging, Freedom, Goodness, Power.

Figure 2 provides an integrated overview of the initial semantic configuration, in the composite visualization background shading represents overall semantic frequency across speakers, while circle sizes indicate frequency of semantic activity for each speaker. The therapist's position within each cell (red circle) is aligned with the family member showing the closest level of semantic activity in that meaning domain, thus allowing therapist's contribution to be read in direct relation to the semantic material currently produced in the interaction.

Figure 2 shows that, across all semantic grids, family members display differentiated intensities of semantic production, with Irene consistently showing high frequencies of re-expressions across grids, most notably in Belonging and Power. The therapist's semantic activity closely follows these configurations: rather than introducing a distinct semantic profile, the therapist's contributions are aligned in proximity to the family member showing a comparable level of semantic activity within each specific semantic field. This happens most often with Irene, the identified patient, with the exceptions of Goodness grid's expression and re-expression, as well as Belonging grid's polarity expression. This suggests an early stance oriented toward engaging with the same levels of expressed NSP and ISP, strategically across specific speakers, while simultaneously operating at the level of meaning re-expression and semantic shift, rather than restructuring the dialogue through independent semantic introductions.

**Figure 3:**
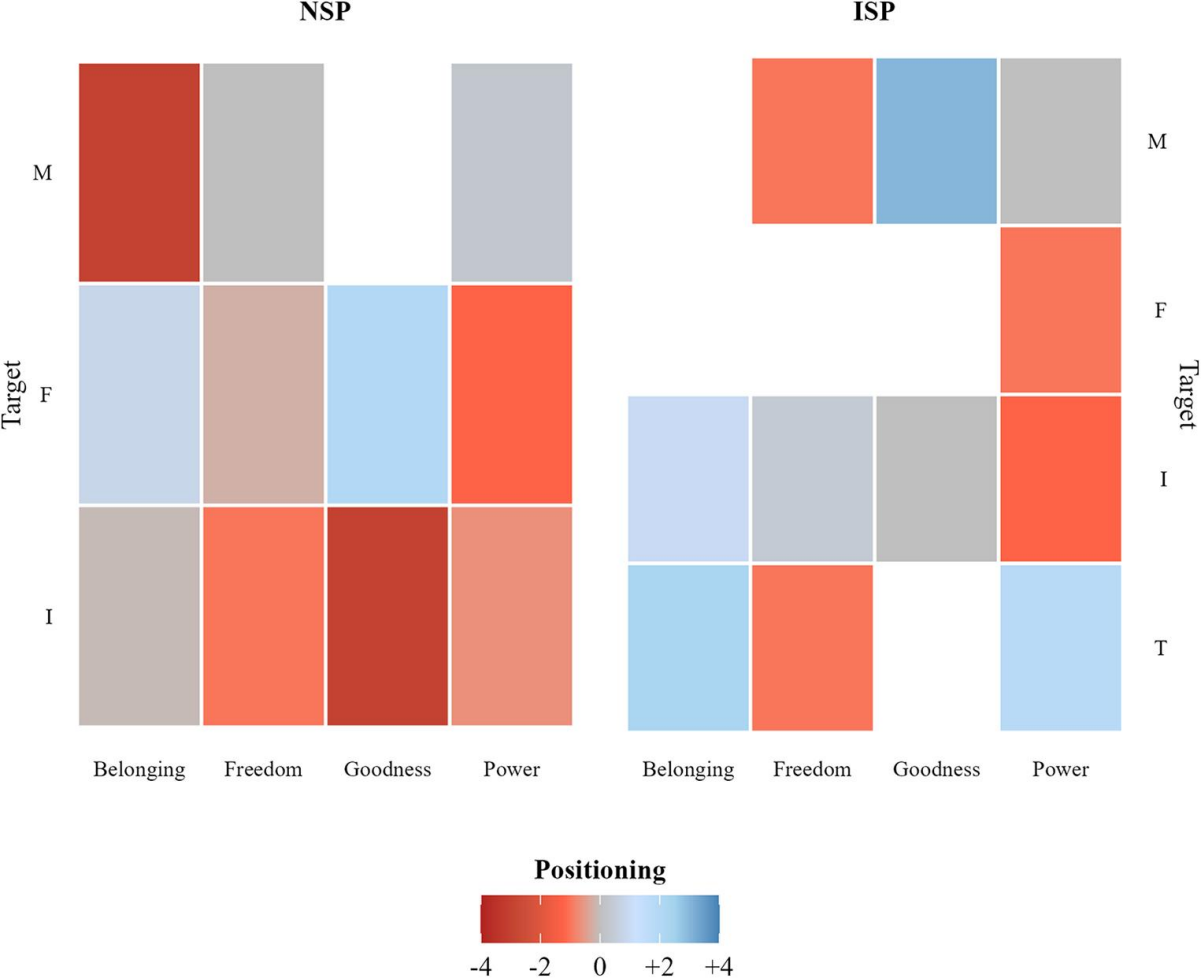
Heatmap: Mother's semantic positioning (initial phase—P1, S1). *Note*. Speaker: I, Irene; F, father; M, mother; T, Therapist.

**Figure 4:**
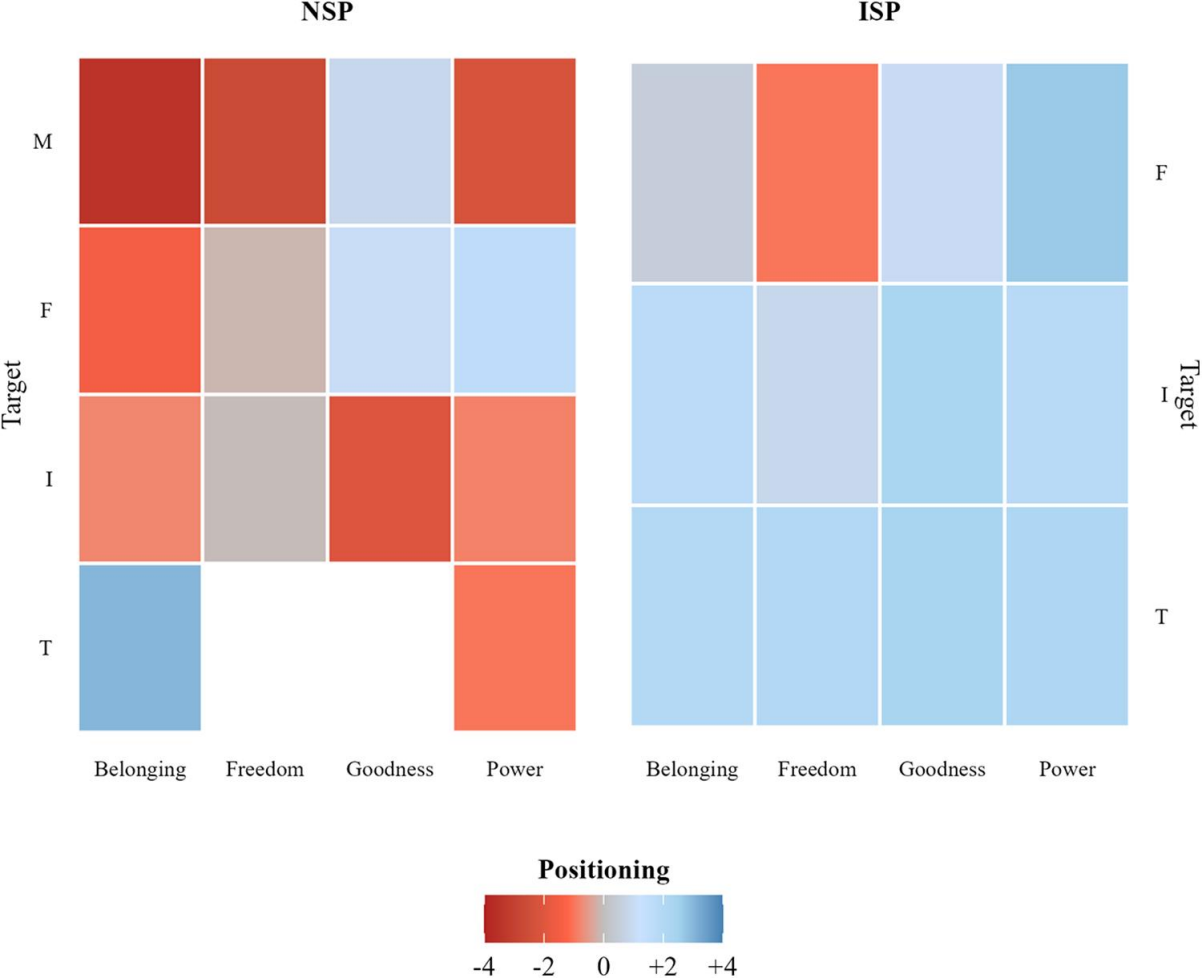
Heatmap: Father's semantic positioning (initial phase—P2, S2). *Note*. Speaker: I, Irene; F, father; M, mother; T, Therapist.

**Figure 5:**
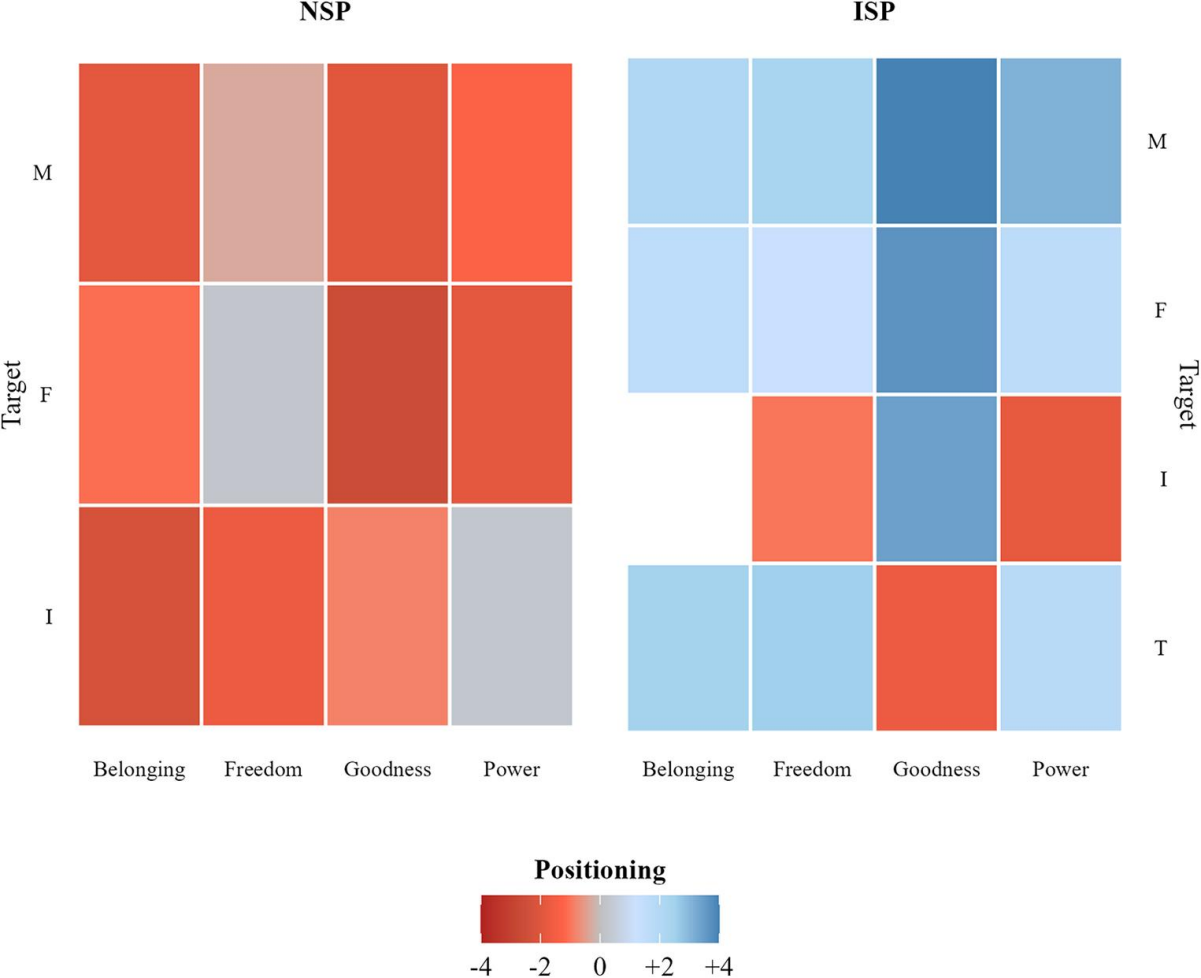
Heatmap: Irene's semantic positioning (initial phase—S1, S2). *Note*. Speaker: I, Irene; F, father; M, mother; T, Therapist.

**Figure 6:**
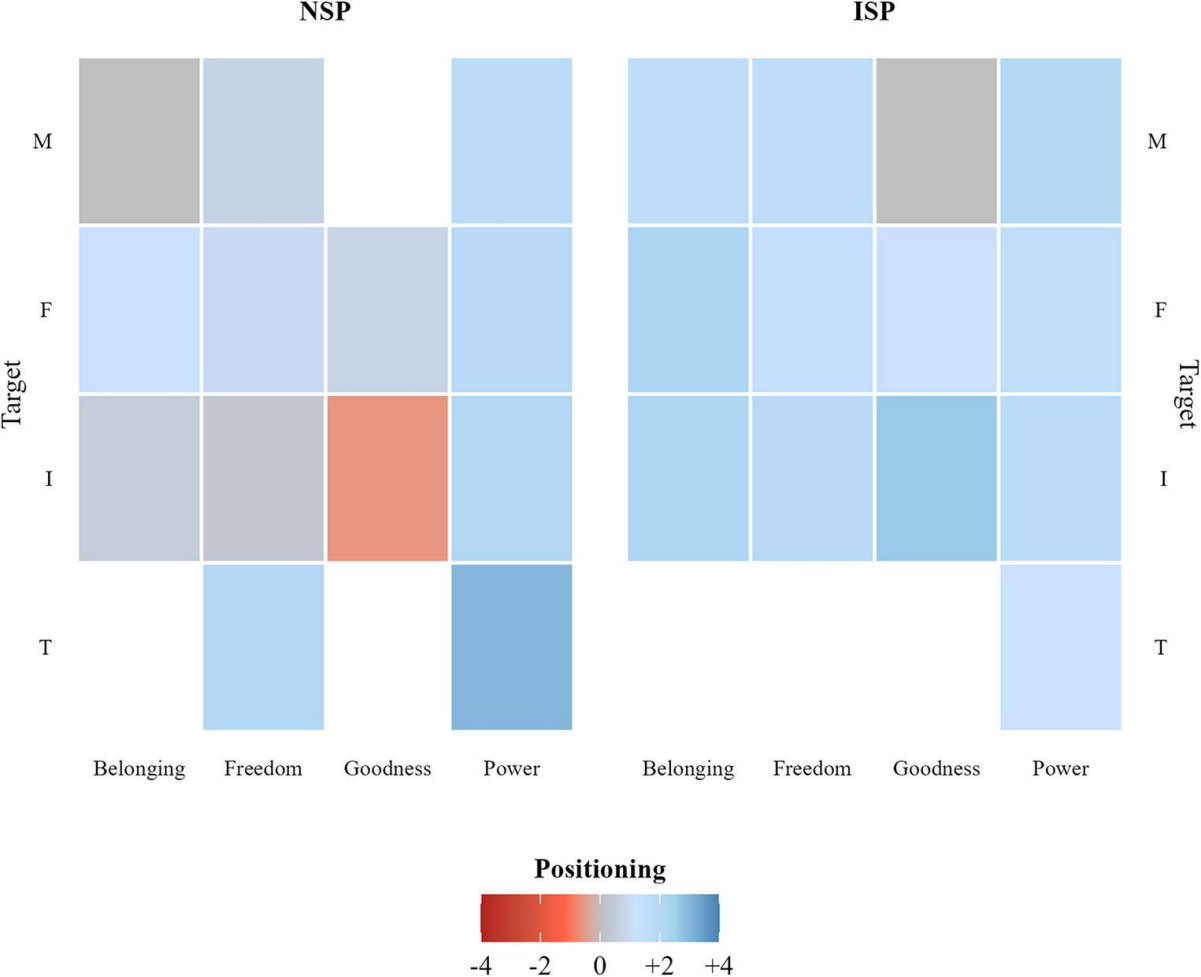
Heatmap: Therapist's semantic positioning (initial phase—P1, P2, S1, S2). *Note*. Speaker: I, Irene; F, father; M, mother; T, Therapist.

To clarify why the therapist shows this kind of activity pattern, semantic profiles through which each speaker describes themselves and the others are presented (cf. Figures 3–6). Positioning is computed as the average signed intensity toward each target person. Accordingly, grey cells indicate orientations close to 0, reflecting either close to zero positive or negative contributions, or a higher proportion of neutral instances. It must be highlighted that some ISP may have as “target” a person who is not physically present when the positioning about that absent person is enacted while speaking to a present person (e.g., through reported speech, relational moves addressed to the therapist, etc.). Following heatmaps show, for each speaker, the mean orientation toward each target (M = mother, F = father, I = Irene, T = therapist) across the four semantic grids, so that NSP and ISP positioning patterns can be compared at a glance for self and others (see Figures 3–6). For each heatmap, we also list, for descriptive purposes, the most salient polarities for that speaker, indicating their value as positive (+), neutral (n), or negative (–).

In NSP production, Belonging portrays the mother's self-positioning as despondent and rejected (see Figure 3); toward the father she swings between inclusion and exclusion, while toward Irene she alternates brief togetherness and sharing with a strongly negative portrayal of her daughter marked by desperation, marginalization, shame, and anger. Freedom is largely fear-driven, with a recurrent pull between approach/opening moves and constraining or distancing definitions—most visible in self- and father-directed cells—and it remains fear-anchored toward Irene, with only occasional cues of autonomy and reassurance. Goodness is relatively sparse and tends to be negative toward Irene, who is mainly framed as a victim. By contrast, Power is built around a repeated fighting frame, occasionally counterbalanced by effort and success, yet still accompanied by undertones of inadequacy and endurance; within this grid the father is mostly positioned as fighting and resisting, with some moments of dominance and withdrawal from confrontation, and Irene again combines fighting struggle with achievement alongside a negative marker of humiliation. It is highlighted that no NSPs target the therapist.

ISPs are more selective and situational: with Irene, the mother shows mainly welcoming and cheerful attunement in Belonging and repeated reassurance in Freedom, with only occasional fear and disorientation; with the therapist, ISPs foreground respect and, especially in Power, alliances, adaptation, and valorization aimed at maintaining a good impression.

Father's NSP self-positioning (see Figure 4) is most clearly negative in Belonging, with a non-random valence pattern in which specific polarities are systematically tied to valence: *χ*^2^(*MC*, 14) = 31.2, *p* = .002, Cramer's *V* = .82 (large effect), dominated by anger and disgrace, with only small positive counterpoints (notably sharing, and ostracizing framed positively). In Goodness, blaming_(+)_ and absolving_(+)_ stand out, alongside a negative self-tone (guilt, victim). In Freedom, the core is negative and fear-based, with only a few positive occurrences around being free and getting close. Power's self-portrait includes self-efficacy_(+)_ and resisting_(+)_, but it is ambivalent around fighting, and it also contains low competence/agency meanings such as passive_(–)_, submissive_(–)_, and inadequate_(–)_, which are mostly framed as due to external constraints. Mother is referred to with negative tones and low-agency across grids (rupture in Belonging, dependence/constraint in Freedom, and disempowered). For Irene, the father mixes brief positives in Belonging with denigration_(–)_ and a split framing of anger_(–/+)_, intended sometimes as positively functional and other times as negatively dysregulated. Goodness is mainly accusatory toward Irene, with partial ambivalence around victimhood_(–/+)_. Freedom combines a single strength_(+)_ cue with an overall dependence/autonomy ambivalence plus clear negative descriptors (closing others out, being distant and unpredictable). Finally, on Power positions her as inferior, also oscillating between mediocre and excellent. The therapist is largely positive in NSPs (welcoming), with only one early, unprompted negative punctuation (mediocre in Power) at the very beginning of the first conversation.

In contrast, father's ISPs are denser and more collaborative: with the therapist they foreground respect and honor, with some provoking embedded in an energizing climate (Belonging), as well as getting close and opening up (Freedom), being also organized around adapting/allying and maintaining a good impression in Power. with Irene, ISPs shift to a supportive but directive stance, combining inclusion and positive affect (Belonging); encouragement, protection, and reassurance in Freedom; a negative judging followed by a relieving dynamic in Goodness; and a strongly directive domineering pattern in Power valued as positive by the father, with only occasional negative interactional punctuations (adapting, surrendering).

In Irene's heatmaps (see Figure 5), Belonging concentrates the sharpest burden in NSPs: in self-positioning it is dominated by desperation and anger, repeatedly coupled with ignoring and being alone, with additional punctuations of disgrace, being discarded, and downfall, while the positive counterweight is limited to togetherness and only occasional cues of being chosen/elected or energetic; consistently, valence is not independent from polarity choice, indicating a systematic coupling between specific Belonging polarities and specific values, *χ*^2^ (*MC*, 9) = 25.1, *p* = .004, Cramer's *V* = .90 (large effect). NSPs describing the mother remain prevalently negative (miserable, angry, despairing tones), whereas toward the father the grid becomes conflictive, where brief positives (sharing/togetherness and a single “deserving”) balance a negative core (desperation, anger, abandoning, resentment, alone, excluded), again with a significant polarity-valence association, *χ*^2^(MC, 12) = 24.1, *p* = .0008, Cramer's *V* = .84 (large effect). Goodness in NSPs is self-focused around a victim position, with ambivalent control-related meanings (controlled/controlling) and other split self-ascriptions and relational movements (bad, letting it all out) alongside a thin positive margin (innocent, upright, good); toward the mother it is nearly absent (an isolated strict note), whereas toward the father it stays mainly accusatory and negative. Freedom remains broadly fear-anchored (fear/scaring) with dependence, disorientation, and risk, and only scattered openings toward autonomy or support; toward the mother it is organized around paired opposites (opening vs. closing; distance vs. closeness, with fear sometimes split in value), and toward the father it becomes mixed through ambivalent distance/attachment configurations around risk and protection. Finally, Power shows the clearest internal ambivalence in NSP self-positioning, where inadequacy coexists with comparably salient competence/achievement meanings (succeeding, effort, self-confidence, success), with further negative punctuations (failing, overbearing moves, shame), and here too valence is not randomly distributed across polarities, *χ*^2^(*MC*, 22) = 39.8, *p* = .009, Cramer's *V* = .84 (large effect); toward the mother, Power is pulled toward conflict (fighting, prevaricating) with only low-intensity positive counterpoints (buckling down, accepting ordinariness), and descriptions targeting the father include negative dominance/prevaricating markers with only limited counterpoints (e.g., authenticity, and a split theme of display). Notably, the therapist does not appear as an NSP target. In ISPs, Belonging shifts toward a more affiliative, repairing stance with both parents. Toward the mother, it is almost entirely made up of including_(+/n)_ and mending_(+/n)_. Toward the father, it is richer and explicitly mixed: strongly positive moves (cheerfulness, including, mending) are counterpointed by negative notes (anger/getting angry, sadness) and occasional provocation_(+)_. This interactional Belonging repertoire also shows a non-random association between polarity and valence, *χ*^2^(*MC*, 14) = 39.5, *p* < .0001, Cramer's *V* = .81 (large effect). Toward the therapist, ISPs remain largely positive (cheerfulness, sharing, respecting, mending), with only marginal negative counter-notes (e.g., sadness, exclusion). Goodness is compact and toward the mother (relieving and repulsing, both positive), but expands toward the father into a dynamic of judging followed by relieving, turning instead predominantly negative toward the therapist through self-related guilt interactional meanings. Freedom's ISPs are primarily regulatory and safety-oriented: toward the mother it centers on reassurance with a few neutral/low intensity alarms and protection moves; toward the father reassurance remains central but is counterbalanced by negative fear/disorientation punctuations alongside opening/closing movements; and with the therapist opening up becomes the main interactional move, accompanied by reassurance and rare alarms. Power ISPs are mostly action-focused and strategic, showing mainly valuing and opposing to the mother, with only a few directive moves. The pattern is instead dominated by domineering and valuing toward the father, along with surrendering that appears with mixed valence, plus occasional inadequacy/embarrassment meanings. Power is broader and mostly collaborative toward the therapist, centered on allying and adapting, with some rare negative emotional notes (inadequacy/embarrassment) and few moves to make a good impression.

As mentioned above, NSPs produced by the therapist (T) are sparse overall and concentrate in a few recurrent polarities across the four grids, with target-specific profiles (see Figure 6). In Belonging, toward the mother it essentially repeats contents concerning desperation_(–/n)_, whereas toward the father also NSPs concerning honor_(+)_ occur. T mainly repeats togetherness and welcoming meanings toward Irene, with neutral and positive values. In Freedom, toward both parents fear is repeated as neutrally valued; with the father, therapist's NSPs cluster also around fragility_(–)_ and risk_(–/n)_, with a positive protective set (reassurance_(+)_ and safety_(+)_). When referring to Irene, therapist centers on NSPs of attachment_(n)_ and risk_(–/n)_, with secondary cues split in value of change_(n)_, fear_(+/n)_, and being close_(+/n)_. In Power, toward the father the few repetitions concern boast_(+)_, excellent_(+)_, and fighting_(–/n)_, while toward Irene NSPs are more frequent and dominated by competence/achievement meanings such as excellent_(+/n)_, succeeding_(+)_/successful_(+)_, self-efficacy_(+/n)_, with strong-willed_(n)_ as a minor recurrent note. Goodness is concentrated on being a victim_(–/n)_ as the only repeated NSP toward Irene, accompanied by self-sacrificing_(n)_ and self-denial_(n)_. The mother is mainly described through relieving_(n)_ others by the therapist, who instead refers to the father mostly through blaming [judging_(+/n)_], alongside self-sacrifice_(n)_ and victim_(n)_ meanings.

Therapist ISPs have a stable welcoming core in Belonging, with—including_(+/n)_, respecting_(+/n)_, and sharing_(+/n)_—directed toward the mother, denser toward Irene, and even more dense toward the father. Ignoring_(+/n)_ appears only occasionally toward the father and Irene. Freedom ISPs remain distance-regulatory and calming: toward the mother they are mostly getting close_(+/n)_ and soothing_(+)_; toward the father, and especially Irene, they expand around reassuring_(+/n)_, exploring_(n)_, and getting close_(+/n)_, with encouraging_(+/n)_ and protecting_(+/n)_ becoming highly recurrent toward Irene and, with both such polarities also targeting the father to a lesser extent. In Power, ISPs are dominated by valuing_(+/n)_ and consistently accompanied by allying_(+/n)_; the polarity-valence association is non-random when targeting both the father, *χ*^2^(MC, 16) = 29.7, *p* = .002, Cramer's *V* = .41 (large effect), and Irene, *χ*^2^(MC, 12) = 27.4, *p* = .007, Cramer's *V* = .37 (large effect). Additional Power repeated ISPs are split in value and mainly concern father and Irene adapting_(–/n/+)_, domineering_(n/+)_, and prevaricating_(n/+)_. Goodness ISPs stay marginal and, where present, are mostly relieving_(+/n)_ toward the father, and attracting_(+)_ Irene.

### 3.2 Toward change: linear mixed-effects models

 Table 5 reports the results of LME predicting speaker narrated and interactive polarities (i.e., value intensity and sign –/n/+) from the therapist's positioning (predictor), controlling for the lagged outcome term and elapsed time (days between sessions). A random intercept for event (phone call or session) was included to account for the clustering of observations within interactional events, thus separating the therapist's effect from session-specific baseline differences.

**Table 5:** Linear Mixed-Effects models (LME) of speaker semantic positioning predicted by therapist positioning.

**Speaker**	**Grid**	**n**	***b***	***SE***	***t***	***df***	***p***	***ICC***	$R_{m}^{2}$	$R_{c}^{2}$	**95% *CI***
											**[low | high]**
I	Freedom	31	.605	.264	2.291	27	.030*	.130	.150	.295	[0.063 | 1.147]
	Goodness	8	−.075	1.747	−.043	4	.968	.026	.050	.073	[−4.925 | 4.776]
	Power	71	.320	.123	2.599	67	.011*	.024	.089	.113	[0.074 | 0.566]
	Belonging	52	.786	.212	3.704	48	<.001***	.282	.300	.625	[0.359 | 1.213]
	**Global**	177	.558	.098	5.658	173	<.001***	.056	.197	.257	[0.363 | 0.753]
F	Freedom	11	1.093	.411	2.660	7	.032*	.156	.476	.645	[0.121 | 2.066]
	Goodness	/	/	/	/	/	/	/	/	/	/
	Power	32	.231	.108	2.143	28	.041*	.495	.220	.992	[0.010 | 0.452]
	Belonging	16	-.025	.347	−.072	12	.944	.087	.100	.189	[−0.781 | 0.731]
	**Global**	71	.375	.150	2.497	67	.015*	0	.084	.084	[0.075 | 0.675]
M	Freedom	10	.921	.365	2.521	6	.045*	.193	.460	.675	[0.027 | 1.814]
	Goodness	/	/	/	/	/	/	/	/	/	/
	Power	18	.502	.211	2.374	14	.032*	.078	.257	.337	[0.048 | 0.955]
	Belonging	/	/	/	/	/	/	/	/	/	/
	**Global**	51	.550	.160	3.442	47	.001**	.133	.197	.368	[0.229 | 0.871]

*Note*. ^*^p < .05.

** *p* < .01.

*** *p* < .001.

Since the predictor is always therapist's mean score of value intensity and sign –/n/+ (positioning), it is not repeated in the table to improve readability.

Speaker, Outcome. Speaker's mean score of value intensity and sign –/n/+ (positioning); I, Irene; F, father; M, mother.

Global, all semantic grids considered together (Belonging, Freedom, Goodness, Power).

*ICC*, intraclass correlation coefficient from the intercept-only model (null model), indexing the proportion of variance attributable to the random grouping structure.

$R_{m}^{2}$, marginal *R*^2^ (variance explained by fixed effects).

$R_{c}^{2}$, conditional *R*^2^ (variance explained by fixed + random effects).

/, non computable.

Across significant models, therapist positioning emerged as a consistent predictor of speaker positioning. Especially for Irene (Freedom, Power, Belonging, and Global), but also for her parents and in the same semantic domains for both (Freedom, Power, plus Global). Notably, the Global outcome was significantly predicted for all three family members, providing the most stable summary signal across targets; *ICC*s and the $R_{c}^{2}$—$R_{m}^{2}$ pattern further indicate that these effects coexist with, and are not reducible to, event-level clustering.

Among all models, the best-performing one was Irene's Global model, which is particularly meaningful given that she was the identified patient. Interestingly, despite being the family member with the lowest global change index, the father yields the single most event-sensitive model in the Power domain. For the mother, significant therapist-mother coupling with small to moderate *ICC*s indicates some event-level heterogeneity but an interpretable fixed-effect signal, overall suggesting that her positioning is responsive to the in-event work (phone call or sessions) while still showing event-contingent baselines.

Accordingly, in the following table (Table 6) we report only the significant LME models, estimated separately for NSP and ISP to disentangle narrated vs. interactional positioning dynamics rather than collapsing them into the global indices used above; importantly, model structure and covariates are identical to the general model, ensuring direct comparability across specifications.

**Table 6:** LME results by polarity type (NSP, ISP): therapist-speaker semantic positioning coupling.

**Speaker**	**Grid**	**Polarity Type**	**n**	***b***	***SE***	***t***	***df***	***p***	***ICC***	$R_{m}^{2}$	$R_{c}^{2}$	**95% *CI* [low | high]**
I	Freedom	NSP	13	.720	.261	2.754	9	.0223*	.248	.643	.947	[0.128 | 1.312]
I	**Global**	NSP	49	.463	.193	2.395	45	.0209*	.141	.260	.420	[0.074 | 0.852]
M	Power	ISP	14	.818	.289	2.828	10	.0179*	.151	.522	.687	[0.174 | 1.463]
M	**Global**	ISP	30	.338	.163	2.072	26	.0483*	.132	.196	.344	[0.003 | 0.674]
F	Belonging	ISP	29	.378	.151	2.503	25	.0192*	.263	.353	.647	[0.067 | 0.688]
F	**Global**	ISP	55	.196	.095	2.056	51	.0449*	.185	.077	.299	[0.005 | 0.387]

*Note*. ^*^p < .05.

Since the predictor is always therapist's mean score of value intensity and sign –/n/+ (positioning), it is not repeated in the table to improve readability.

Speaker, Outcome. Speaker's mean score of value intensity and sign –/n/+ (positioning); I,  Irene; F, father; M, mother.

Global, all semantic grids considered together (Belonging, Freedom, Goodness, Power).

NSP, Narrated Semantic Polarities.

ISP, Interactive Semantic Polarities.

*ICC*, intraclass correlation coefficient from the intercept-only model (null model), indexing the proportion of variance attributable to the random grouping structure.

$R_{m}^{2}$, marginal *R*^2^ (variance explained by fixed effects).

$R_{c}^{2}$, conditional *R*^2^ (variance explained by fixed + random effects).

As can be seen in the table, significant therapist-speaker positioning coupling emerged in a select set of NSP and ISP outcomes. Significant effects were observed in Irene's NSP global positioning, specifically in the Freedom domain, with moderate *ICC*s and comparatively higher explained variance ($R_{m}^{2}$), suggesting that her explicit positioning shows a stable linkage with therapist positioning beyond session-specific baselines. For both parents, significant effects were confined to ISP and were visible in global positioning, with modest *ICC*s indicating a coupling that is more clearly expressed at the interactional level. There was a clearer fixed-effect contribution in Power for the mother, and in Belonging for the father, pointing to domain-specific interactional meanings sensitivity in how each parent aligns with, and responds to, the therapist's positioning.

Notably, across both sets of LME results (Tables 5, 6), all significant therapist-speaker positioning coupling effects were positive in direction, indicating alignment tending to positive positioning values whenever an effect was detected.

## 4 Discussion

The present pilot study pursued three tightly linked objectives: (a) to develop and test a rule-based, partially automated workflow for coding clinical narratives through Family Semantic Grid (FSG) framework without losing meaning; (b) to analyze coded semantic polarities emerged during therapeutic dialogue; and (c) to map conversational markers of process-level effectiveness across sessions.

For what concerns objective (a), results support feasibility since the program facilitated the coding phase and yielded analyzable outputs, enabling both descriptive mapping and inferential testing (rank-based contrasts, *χ*^2^-tests, and LME models). The analytic objects subsequently inferred from data, such as “positioning” and “semantic status,” proved coherent enough to sustain different converging analyses. Program's automation stabilized and supported the coding pipeline in a human-in-the-loop procedure (85, 86) without replacing clinician's interpretation, and the kind of data retrieved proved to be a good basis for the analyses (objective b). It is important to underline that the utility of the program lies in supporting the clinician in orienting attention to recurring semantic markers. In fact, it does not work as a substitute for the therapist's intuitive and interpretative abilities, since meanings are still coded individually by the clinician-coder, who brings indispensable expertise and contextual understanding.

Results show that early consultation represents a critical window for the therapist to capture the family's meaning organization and to join the system, not merely as a routine introductory step, but in a way that already sets the conditions for the work that follows. In this sense, the consultation is a high-yield phase for assessment and hypothesis-building prior to formal contracting (105). In fact, the therapist's moves are not inconsequential (cf. Figure 2), and the present findings suggest they are already highly specific in how they keep the semantic field workable while entering the family's ongoing organization of meanings (objective b). In fact, across early events, a clear and theoretically meaningful separation between NSP and ISP was visible: ISPs were generally more positively oriented, while NSPs tended to be lower or negative—and more dispersed—with several non-normal distributions. This finding suggests that “what is said about” (narrated meanings) and “what is done” (interactive meanings) form partially distinct semantic layers, and this is consistent with what has been reported in previous literature [FSG; (72–75)]. The semantic heatmaps further show a differentiated “semantic ecology” rather than a single shared negative narrative: Irene showed the most split profile across grids, consistent with the difficulty to find a coherent positioning in her original conversational context (56, 106), while the therapist displayed a broadly positive, comparatively stable positioning across domains (107). Importantly, when moving from how meanings are presented (NSP) to how meanings are enacted (ISP), the semantic-status analyses converged on a robust pattern: in line with Ugazio et al. (108), the therapist showed fewer first expressions and more re-expressions and semantic shifts if compared with each family member, i.e., an interactional stance organized around uptake, reworking, and direction change rather than semantic imposition, consistent with a congruent (109)—not collusive—way of working within the system's initial meaning organization. This is a clear process-level result: it operationalizes a strategy compatible with an early alliance (110), such as working “inside” the family's activated semantic material. Thus, the therapist's mostly positive orientation—which does not imply justifying—across grids is consistent with an early generative framing that protects alliance conditions by keeping the semantic field workable even when negative narrated or interactive content is engaged (111), while simultaneously building the informational and hypothesizing basis (e.g., family history, relational dynamics, shared definitions) for a later feedback that can re-situate or, even totally reverse, key meanings. This therapist positioning and interactive moves may be interpreted as functional to *in vivo* micro-transformations of meanings introduced by family members. Narrated and interactive meanings are taken up and reflected back in a more positive and acceptable sense and, conversely, those meanings or moves carrying negative implications—yet framed by the speaker as good or justified—are met without direct confrontation and worked toward neutrality (or a constructive reversal). This early meaning management and uptake is strategically tailored for the specific system—therefore more readily incorporated into it—and culminates in a more explicit perturbing (112) but plausible (113) reversal, conceptualizable as a destabilizing jolt that disrupts an entrenched meaning pattern and facilitates transition toward a more functional alternative (114). Such reversal happens mainly during the feedback session, which signs the end of the consultation phase and the formal beginning of the system's engagement toward shared objectives (i.e., therapeutic work).

The longitudinal LME models, addressing objective (c), include also data from the feedback sessions and extend the descriptive picture by testing whether therapist positioning predicts speaker positioning beyond short-term autocorrelation (lag) and elapsed time, while accounting for event clustering. Across the general LMEs (Table 5), the significant effects were consistent and moving toward positive values: therapist positioning consistently predicted global speaker positioning, with the clearest and most robust pattern for Irene, whose Global index provided the most stable summary signal; *ICC* and $R_{c}^{2}$*—*$R_{m}^{2}$ pattern combinations suggest that the effect coexisted with event-level clustering, not being absorbed by it. This supports multilevel specification while also pointing to a stable linkage between therapist and Irene (identified patient) across events. By contrast, the father showed higher *ICC* in Power domain, which indicates that this is the most event-sensitive model, with his positioning in this semantic varying markedly across events. However, since coupling with therapist positioning is still significant—and changing in time toward positive semantic values—this result is consistent with considering session context as a key driver of moments of re-positioning in Power. Concerning the mother, small to moderate *ICC*s appear along with significant effects, indicating both event-contingent baselines and fixed-effect evidence, consistent with a positioning that remains overall responsive, especially during in-event interactions. A more selective and polarity-specific pattern became visible when NSP and ISP dynamics were observed separately (Table 6); for Irene, significant effects emerged in global NSP and in Freedom grid, that for her revolves around autonomy and relational-distance regulation themes. For both parents, interestingly, significant effects were confined to ISP outcomes at the Global level for both, plus a differentiation of effects in Power for the mother and Belonging for the father. This indicates that, for parents, alignment with therapist positioning primarily happens through ISPs. Moreover, the specific domain may differ from the one that is observed as locus of change in NSP, in fact father positioning did not show an effect in Belonging when NSP and ISP were considered together (cf. Table 5). This concretely shows that the parents have been successfully approached by therapist being with them in the interaction, and genuinely (109), acting different ISPs, rather than by verbally correcting their NSPs.

Overall, the general LME models (Table 5) show that significant effects were all positive in direction for each family member, consistently indicating alignment with therapist positioning. The few non-significant estimates and non-estimable cells were confined to sparse combinations of speaker, grid, and FSG type. Such cells should be read as limited information rather than counterevidence, reflecting domains that were marginally engaged by the therapeutic work in this phase. This is visible in the general LME models for Goodness, where both parents were not estimable and, consistently, this is the only domain in which Irene did not show a significant effect.

To the best of our knowledge, this is the first study to combine a rule-based, partially automated FSG coding pipeline with multilevel (mixed-effects) modeling to quantify therapist-family semantic positioning dynamics, separately for NSP and ISP. Results further suggest that the adopted methodology is well-suited to capture and quantify process-level therapeutic dynamics within the systemic-relational framework of Semantic Polarities Theory. Moreover, within the boundaries of a single-case design, it is still legitimate to note convergence between process markers and clinically salient milestones: the overall pattern of positive-direction coupling in significant models, together with early therapist emphasis on re-expression/shift, and feedback session co-occurred with subsequent clinically meaningful developments. This quantitative pattern is coherent with key clinical milestones observed over the same period and reported here only as qualitative contextual convergence, including the remission of Irene's suicidal ideation, the absence of reported self-harming behavior, and the progressive attainment of a shared setting for all family members in S12, despite the high interparental conflict.

Taken together, these results may suggest that process-relevant information can be retained even if clinical disclosure remains minimal: since the analytic focus is placed on positioning dynamics, the analyses of the present work produced a sort of “semantography” in that they provide a compact, data-grounded mapping of interpretable meaning patterns across interactional therapeutic events.

### 4.1 Limitations and future directions

It is important to read this study in light of its limitations. First, this is a single-case pilot, and the generalizability of the observed patterns is necessarily limited. Second, the procedure is lexicon-dependent and relies on human-in-the-loop decisions: coverage and potential bias depend on the lexical library and synonym expansion. Further, the present application is restricted to the nuclear family, without extending to significant others and/or the broader family system; further analyses may benefit from including these levels in future analyses. An important limit concerns the fact that the current implementation is not user-friendly yet: the program runs in a Python development environment, which currently limits accessibility outside programming settings.

Although the single-case pilot design allowed us to test the feasibility of the procedure, it necessarily limits its general validity. Future work should therefore apply this procedure to multiple cases simultaneously, preferably across different symptomatic presentations, or control groups. This would allow for comparison analyses across conditions. Alongside this, a complementary direction would be to compare the present procedure with existing tools for transcript-based analysis, such as DAAPLab© (115).

Another relevant development would be to examine symptom trajectories in light of changes in semantic patterns. This could be supported by the “extra-semantic referents” additional output (cf. Supplementary Material), which includes clinically relevant elements not directly codifiable within the FSGs, such as symptoms, affective states, specific manifestations of neurodevelopmental conditions, or significant life events.

Ideally, multi-therapist corpora of therapeutic work with similar clinical situations would add value, since it would allow testing whether meaning patterns change due to therapist-specific effects. Moreover, including sessions from the final phase of therapy would extend the longitudinal window, allowing for the exploration of early patterns within longer trajectories of change. It would be also of great interest to compare ISPs between online and in-person sessions, since online therapies are widespread nowadays.

A practical priority would be to package the program into a more user-friendly tool and implement a more intuitive interface, to support broader and more consistent adoption beyond programming environments. Future studies could also consider adding machine learning to the current program, to further improve transcript coding.

## 5 Conclusions

Taken together, these findings indicate that a partially automated, rule-based text-mining workflow can be applied to FSG coding and can yield process indicators that remain clinically legible. In this case, the early consultation phase was marked by a clear differentiation between NSPs and ISPs, a therapist semantic stance centered on re-expressions and shifts, and consistently positive therapist-speaker coupling, globally most robust for the identified patient, and polarity-specifically differentiated between Irene (NSP) and the parents (ISP). Read in this way, the analysis provides a compact semantography of the case: a data-grounded mapping of coupling and change that can inform process interpretation while remaining parsimonious on case-specific disclosure.

Besides the program development, immediate practical implications of the present work include providing a replicable analysis procedure to be applied also to other meaning indicators outside Semantic Polarity Theory domain; showing useful insights for clinical practice; and contributing to therapist training resources through a quantitative, yet case-focused, lens.

## Data Availability

The datasets presented in this article are not readily available because clinical case data can not be shared. Requests to access the datasets should be directed to eleonora.florio@guest.unibg.it.
