# Supplementary material for: Toward the automation of family semantic grids: a pilot exploration of text mining for evidence-informed systemic-relational psychotherapy
Source: Front Child Adolesc Psychiatry. 2026 Jul 23;5:1800848. doi: 10.3389/frcha.2026.1800848 (PMC13443033; doi:10.3389/frcha.2026.1800848)
Supplement: Supplementary file 1 [file Supplementaryfile1.pdf]

Supplementary Material

1 Family semantics and their original operationalization

It is useful to briefly outline the four main family semantics identified by Ugazio – freedom, goodness, power, and belonging. These represent the principal conversational fields through which individual experiences and relational patterns are organized, yet they rarely appear in pure form in reality. However, they have been found to prevail in clinical settings respectively in phobic, obsessive-compulsive, eating and feeding, and depressive disorders (Ugazio, 1998, 2013; Ugazio et al., 2009, 2011); therefore, it is appropriate to outline them all following Ugazio's elaboration (Ugazio, 1998, 2013; Ugazio et al., 2009) before approaching the clinical case.

The semantics of freedom revolves around two main polarities: "freedom - dependency" and "exploration - attachment." The tension is between autonomy and emotional bonds, which may be felt as limiting precisely because they are conceived as protective. In this semantic field, independence, exploration, and risk tend to be valued positively, while attachment and the search for safety often take on a negative tone, as signs of fear or dependency. The resulting conversational pattern oscillates between closeness and distance: the person may feel constrained in a close relationship –experienced as protection – and frightened yet free when alone. The dominant emotions are courage and fear, and the prevailing relational logic concerns the negotiation of distance and reassurance, seeking to balance an acceptable level of self-esteem with connection and safety within relational closeness.

At the core of the semantics of goodness lie the two polarities "good (abstemious) - bad" and "dead - alive." "Goodness" in this semantic field is equated with abstinence and self-sacrifice, whereas everything that pertains to vitality, pleasure, and personal gratification tends to be considered "bad". In this moral order, renunciation and purity are valued over desire and self-gratification: chastity and sacrifice become signs of moral worth, while pursuing impulses and desires denotes selfishness and is considered nasty. Indeed, guilt, virtue, and moral rigor are the core themes in conversations, and the emotional climate is dense with shame, guilt, and a sense of innocence. The main relational movements therefore are "abstaining" and "self-sacrificing" versus "corrupting" and "taking advantage," where the former imply withdrawal but are valued positively, while the latter denote exchanges with others that are morally condemned.

"Winner - loser" and "strong-willed - yielding" polarities, characterize the semantics of power. This is a profoundly relational and comparative field because in these families identity depends directly on others' recognition and appraisal, which are central to self-definition. Therefore, external judgements and success become focal points and there is more involvement in competition rather than cooperation, with individuals struggling for dominance in relationships and in life to the point that what matters is not the content of conflict but who prevails in it. As a result, differences among individuals are never neutral and become hierarchies of value, with self-esteem fluctuating according to victory (which fosters a sense of competence, efficacy, and self-trust) or defeat (accompanied by feelings of inadequacy and impotence). Concerning the typical ways of relating, those who prevail tend to conform to those in even higher positions in order to preserve their status, while those who perceive themselves on the losing pole feel unable to improve their standing and react by opposing the winners, attempting to undermine their superiority.

Finally, the semantics of belonging is articulated across the polarities "inclusion - exclusion" and "honor - disgrace" with the core concern whether individuals are included and welcomed or cast out from their group – e.g., family, community, or lineage. In this sense, inclusion is an honor because confirms one's worth, while exclusion entails loss of dignity and, in the long run, disgrace. This semantic field is characterized by a complex moral economy in which, for example, belonging can also be resisted when it comes into conflict with personal moral integrity. The prevailing emotions are gratitude and joy when acceptance is experienced, anger and despair when felt rejected, and resentement when inclusion is obtained at the cost of personal dignity. Consistently, "including - ostracizing" represents represents a common way of relating within the semantics of belonging, and the excluded person is not necessarily despised: one can, for instance, be honorable yet remain unacknowledged.

These four family semantics have been subsequently operationalized for research purposes through the Family Semantic Grid (FSG) in its different versions (Ugazio et al., 2009, 2018; Ugazio & Castelli, 2015; Ugazio & Guarnieri, 2017), which allow the identification of the dominant meanings and conversational positions within family narratives (Narrated Semantic Polarities; NSP) and in-person interactions (Interactive Semantic Polarities; ISP). This theoretical framework serves as the conceptual basis for the present study and its analytic aims.

2 Family Semantic Grids

In this section original tables of Family Semantic Grids are provided. Following tables are retrieved and adapted from (Ugazio et al., 2009, 2018; Ugazio & Castelli, 2015; Ugazio & Guarnieri, 2017) and refer to Narrated Semantic Polarities (NSP) and Interactive Semantic Polarities (ISP).

Table 1. Semantics of Freedom grid (NSP)

**VALUES**	
FREEDOM	DEPENDENCY
EXPLORATION	ATTACHMENT
RISK	SAFETY
CHANGE	STABILITY
**DEFINITIONS OF SELF/OTHERS/RELATIONSHIPS**	
FREE	DEPENDENT
Self-sufficient	Conditioned by others
Explorative	Trapped
Unattached	Bound
UP AGAINST THE ODDS	PROTECTED
Nomadic	Sedentary
Precarious	Stable
Disoriented	Safeguarded
UNPREDICTABLE	RELIABLE
Distant	Close
Stranger	Familiar
Dangerous	Reassuring
COURAGEOUS	FEARFUL
Rash	Cautious
Careless	Careful
Bold	Cowardly
STRONG	WEAK
Invulnerable	Fragile
**WAYS OF RELATING**	
KEEPING DISTANT	GETTING CLOSE
Counting on oneself	Counting on others
Opening to others	Closing others out
GETTING FREE FRON OTHERS	DEPENDING ON ITHERS
Breaking free	Clinging to others
Keeping self-sufficient	Relying on others
EXPLORING	STAYING PUT
Opening to novelty	Digging in
Taking risks	Protecting oneself
SCARING	REASSURING
Disorienting	Guiding
Alarming	Calming
**EMOTIONS AND FEELINGS**	
COURAGE	FEAR
Disorientation	Constraint

Table 2. Semantics of Goodness grid (NSP)

**VALUES**	
ABSTEMIOUS GOODNESS	EVIL
CHASTITY	DEPRAVITY
SELF-DENIAL	SELF-ASSERTION
HOLINESS	WICKEDNESS
**DEFINITIONS OF SELF/OTHERS/RELATIONSHIPS**	
GOOD (ABSTEMIOUS)	BAD
Self-sacrificing	Selfish
Sparing	Greedy
Responsible	Careless
UPRIGHT	IMMORAL
Abstemious	Pleasure-seeking
Spiritual	Carnal
Chaste	Depraved
STRICT	PERMISSIVE
Repressed	Instinctive
Controlled/-ing	Spontaneous
Stingy	Spendthrift
INNOCENT	GUILTY
Mortified	Thirsty for life
Disgusted	Sated
Victim	Torturer
DEAD	ALIVE
clean	Dirty
**WAYS OF RELATING**	
ABSTAINING	CORRUPTING
Renouncing (sex)	Enjoying (sex)
Cleaning	Dirtying
SELF-SACRIFICING	TAKING ADVANTAGE
Feeling responsible	Not giving a damn
Depriving oneself	Exploiting others
REDEEMING	DEPRAVING
Repressing	Letting it all out
Forbidding	Transgressing
CONDEMNING	ABSOLVING
Blaming	Scandalizing
Amending	Sinning
**EMOTIONS AND FEELINGS**	
INNOCENCE	GUILT
Disgust	Pleasure

Table 3. Semantics of Power grid (NSP)

**VALUES**	
SUCCESS	FAILURE
POWER	SUBMISSION
DETERMINATION	COMPLIANCE
DISPLAY	AUTHENTICITY
**DEFINITIONS OF SELF/OTHERS/RELATIONSHIPS**	
WINNER	**LOSER**
Successful	Defeated
Excellent	Mediocre
Superior	Inferior
STRONG-WILLED	YIELDING
Efficient	Inept
Gritty	Weak-willed
Competitive	Spine-less
OVERBEARING	SUBMISSIVE
Assertive	Meek
Challenging	Retiring
Imposing	Complying
BOASTFUL	HUMBLE
Arrogant	Laid back
Self-confident	Ill at ease
Double-faced	Authentic
GOOD-LOOKING	UGLY
Slim	Fat
**WAYS OF RELATING**	
ADAPTING	RESISTING
Gaining consent	Stepping off
Prevaricating	Putting up with
WINNING	LOSING
Succeeding	Failing
Excelling	Accepting ordinariness
FIGHTING	SURRENDERING
Buckling down	Giving in
competing	Withdrawing
MAKING AN IMPRESSION	MAKING A POOR IMPRESSION
Boasting	Self-Abasing
Patronizing others	Cutting others down to size
**EMOTIONS AND FEELINGS**	
BOAST	SHAME
Self-efficacy	Inadequacy

Table 4. Semantics of Belonging grid (NSP)

**VALUES**	
INCLUSION	EXCLUSION
HONOR	DISGRACE
BEING CHOSEN	BEING REJECTED
GLORY	DOWNFALL
**DEFINITIONS OF SELF/OTHERS/RELATIONSHIPS**	
IN THE GROUP	OUT OF THE GROUP
Belonging	Excluded
Being welcomed	Being discarded
Accepted	Kept out
WORTHY	UNWORTHY
Respectable	Contemptible
Honorable	Despicable
Deserving	Reprehensible
ELECTED	OUTCAST
Rewarded	Deprived
Respected	Refused
Revered	Defrauded
GRATEFUL	ANGRY
Enthusiastic	Miserable
Joyful	Inconsolable
Merry	Hopeless
ENERGETIC	RUN DOWN
Together (with)	Alone
**WAYS OF RELATING**	
INCLUDING	OSTRACIZING
Sharing	Cutting off
Welcoming	Abandoning
HONORING	DISHONORING
Deserving	Usurping
Ennobling	Discrediting
OVERWHELMING WITH GOODS	DEFRAUDING
Remembering	Forgetting
Celebrating	Ignoring
VENERATING	DESTRYING
Enthusing	Getting down
Jubilating	Regretting
**EMOTIONS AND FEELINGS**	
JOY	DESPERATION/ANGER
Gratefulness	Resentment

Table 5. Interactive Semantic Polarities (ISP) grid

**SEMANTIC OF FREEDOM**	
Getting close	Keeping distance
Opening up	Closing up
Encouraging	Limiting
Exploring	Staying put
Reassuring/Protecting	Alarming
Taking risks	Getting scared
EMOTIONS AND FEELINGS	
Courage	Fear
Disorientation	Constraint
**SEMANTIC OF GOODNESS**	
Restraining	Unlashing
Resisting	Tempting
Self-sacrificing	Taking advantage
Being responsible/guilty	Shrugging off
Judging	Relieving
Repulsing	Attractive
EMOTIONS AND FEELINGS	
Disgust	Pleasure
Mortification/Innocence	Guilt
**SEMANTIC OF POWER**	
Fighting/Domineering	Surrendering
Competing	Allying
Valuing	Criticizing
Adapting	Opposing
Making an impression	Making a poor impression
Bragging/Humiliating	Being humiliated/Embarassing
EMOTIONS AND FEELINGS	
Disgust	Pleasure
Mortification/Innocence	Guilt
**SEMANTIC OF BELONGING**	
Sharing	Ignoring
Including	(Self-)Excluding
Worshipping/Celebrating	Destroying
Mending	Provoking
Respecting/Honoring	Dishonoring
Rejoicing/Energizing	Getting angry
EMOTIONS AND FEELINGS	
Joy	Anger
Cheerfulness	Sadness/Despair

3 Program construction specifications

Having the software the same structure of narrative and interactive FSGs, enriched with synonyms traceable to the original lexical items of the FSGs, the original hierarchical structure (i.e., grids, sub-grids, and semantic polarities) was preserved. The transcripts must be in the plain-text (.txt), and each speaker's turn is on a separate line, so that line numbering corresponds directly to turns of talk. Interruptions were coded by appending the numeral "1" to the speaker label (e.g., F1 = interruption by the father), a convention that was recognized by the program as referring to the same speaker. Speakers are identified by their position at the beginning of the line, and upon opening a transcript the program prompts the user to select the speaker to be analyzed; nevertheless, surrounding turns by other speakers are retained and displayed to support contextual interpretation within the broader conversational flow.

Detected terms are presented to the therapist highlighted within their textual context and associated with their line number; surrounding segments without automatically detected terms are also displayed to support contextual monitoring and the identification of clinically relevant meanings not initially captured by the program, including those affected by possible transcription errors.

Although the addition of entirely new semantic polarities was technically possible, it was not required in this study, as this preliminary validation phase of the program was deliberately conducted in close adherence to the original narrative and interactive polarities of FSGs (Ugazio et al., 2009, 2018), which were fully retained in their original structure. But, in order to facilitate the extraction of clinically relevant information through the software, a set of additional functionalities was implemented at the program level, with the aim of generating outputs useful for systematic analysis rather than altering the semantic framework itself.

A first relevant difference concerns the generation of an additional output file, labeled "extra-semantic referents" (not analyzed in the present study), which collects those terms that are not directly codifiable within the FSGs but refer to relevant elements in connection with the codified meanings. Such referents include psychological symptoms (e.g., anxiety or depressive states), affective or emotional references not represented within the grids (e.g., love or affection), as well as specific conditions (e.g., ADHD) or significant life events.

The choice of a continuum ranging from -4 to +4 to intensity differences represents a further difference at the level of data entry, since in the original FSG coding procedure, semantic positioning is expressed through ordinal categories – Pole 1, Intermediate 1, Median, Intermediate 2, and Pole 2 (Ugazio et al., 2009; Ugazio & Guarnieri, 2017) – which are clinically meaningful and are here adapted to meet the requirements of systematic computational analyses. In fact, for the purposes of the present study, this ordinal structure was translated into a signed numerical range from -4 to +4, in order to support the analysis of semantic distribution, co-occurrence, and variation across conversational turns. The program, beside prompting the coder to specify both the referent to whom the meaning applies and the speaker producing it, shows also a field to specify the response modality (confirmation, disconfirmation, rejection, or correction); confirmation is set as the default option to reflect the expected flow of conversation and to optimize data entry, while alternative response modalities request direct selection. Therefore, the clinician can confirm or revise lexical allocations within the grids through structured menus, assign polarity and intensity values, and introduce new lexical items or extra-semantic referents when needed. A last difference worthy of note is that in the original FSG coding procedure (Ugazio et al., 2009), semantic polarities were classified within narrative units following the criteria of the Narrative Process Coding System (Angus et al., 1999), consistent with the analytic strategy focusing on topic-based units and further distinguishing polarities within them as first expressions, re-expressions, or semantic shifts.
